# Attenuated DNA damage repair delays therapy-related myeloid neoplasms in a mouse model

**DOI:** 10.1038/cddis.2016.298

**Published:** 2016-10-06

**Authors:** Kit I Tong, Kazushige Ota, Akiyoshi Komuro, Takeshi Ueda, Akihiko Ito, C Anne Koch, Hitoshi Okada

**Affiliations:** 1 The Campbell Family Institute for Breast Cancer Research, Ontario Cancer Institute, University Health Network, Toronto, ON, Canada M5G 2M9; 2 Department of Biochemistry, Kindai University Faculty of Medicine, 377-2 Ohno-Higashi, Osaka-Sayama 589-8511, Osaka, Japan; 3 Department of Pathology, Kindai University Faculty of Medicine, 377-2 Ohno-Higashi, Osaka-Sayama, Osaka 589-8511, Japan; 4 Radiation Medicine Program, Princess Margaret Cancer Center, University Health Network, Toronto, ON, Canada M5G 2M9; 5 Department of Medical Biophysics, University of Toronto, Toronto, ON, Canada M5G 2M9; 6 Anti-Aging Center, Kindai University, Higashi-Osaka, Osaka 577-8502, Japan

## Abstract

Therapy-related cancers are potentially fatal late life complications for patients who received radio- or chemotherapy. So far, the mouse model showing reduction or delay of these diseases has not been described. We found that the disruption of *Aplf* in mice moderately attenuated DNA damage repair and, unexpectedly, impeded myeloid neoplasms after exposure to ionizing radiation (IR). Irradiated mutant mice showed higher rates of p53-dependent cell death, fewer chromosomal translocations, and a delay in malignancy-induce;/– mice. Depletion of APLF in non-tumorigenic human cells also markedly reduced the risk of radiation-induced chromosomal aberrations. We therefore conclude that proficient DNA damage repair may promote chromosomal aberrations in normal tissues after irradiation and induce malignant evolution, thus illustrating the potential benefit in sensitizing p53 function by manipulating DNA repair efficiency in cancer patients undergoing genotoxic therapies.

In USA alone therapy-related cancers, either hematological or solid, among cancer survivors account about 18% of all incident cancers, surpassing primary cancers of the breast, lung, and prostate.^1^ Therapy-related myeloid neoplasms (t-MNs) include therapy-related myelodysplastic syndrome (t-MDS) and acute myeloid leukemia (t-AML). Patients with t-MDS or t-AML have similar survival rates while patients with complex and unfavorable karyotypes tend to have poorer prognosis regardless of morphologic presentation and myeloblast percentage.^2^ Mutations in *TP53* and *ATM* (ataxia telangiectasia mutated) are considered risk factors due to their important roles in the DNA damage response (DDR) pathways.^3, 4^ At present there is no active medical intervention to prevent the risk of these therapy-induced cancers. In addition, mouse models that demonstrate a delay of therapy-related malignancies have not yet been described.

Genotoxic therapies generate DNA damage, including potentially deleterious DNA double-strand breaks (DSBs) that may be repaired by non-homologous end joining (NHEJ).^5, 6^ NHEJ does not utilize extensive homology for DSB end joining and is active throughout the entire cell cycle. NHEJ consists of the classical (C-NHEJ) and alternative (A-EJ) NHEJ pathways.^6, 7^ The C-NHEJ pathway enables direct joins and low microhomology usage (1–3 nucleotides (nt)) at DSB repaired junctions, and A-EJ prefers longer microhomology-mediated end joining (≥4 nt) at ligation sites.

Aprataxin and PNKP-like factor (APLF, also referred to as Xip1, C2orf13, and PALF) is a poly(ADP-ribose) or PAR-binding protein that interacts with C-NHEJ repair factors, XRCC4-DNA ligase 4 and Ku, to facilitate C-NHEJ in a PAR polymerase 3 (PARP3)-dependent manner.^8, 9, 10^ APLF can undergo ATM- and PARP3-dependent phosphorylation at serine-116 following ionizing radiation (IR), which is critical for the recruitment of APLF to the sites of DNA lesion to resolve *γ*H2AX DNA damage signals.^11^ Accumulation of APLF to DSBs has been demonstrated to promote the retention of XRCC4/DNA ligase 4 complex in chromatin to facilitate DNA ligation during C-NHEJ.^10^ Furthermore, a phospho-ablative mutant of APLF (APLF^S116A^), which disabled IR-induced phosphoryation at serine-116 of APLF, exhibited higher persistent *γ*H2AX DNA damage signal and lower cellular survival in colony formation assays after IR exposure reminiscent of cells with APLF depletion.^11^ These data suggest that APLF works downstream of ATM and PARP3 to modulate C-NHEJ after IR treatment.

Ionizing radiation and reactive oxygen species (ROS) generate DSBs with chemically modified ends, which require DNA end processing before end joining. Nucleases such as Artemis and APLF, and end processing enzymes such as PNKP (polynuceotide kinase/phosphatase), may participate in processing IR- or ROS-damaged DNA ends for DNA damage repair.^6^

Here we report that a moderate reduction of C-NHEJ activity by the depletion of APLF impedes radiation-induced oncogenic translocation in normal tissues and malignancy-associated mortality.

## Results

### *Aplf*^–/–^ mice are moderately impaired in C-NHEJ activity without a switch to A-EJ

**Figure 1:**
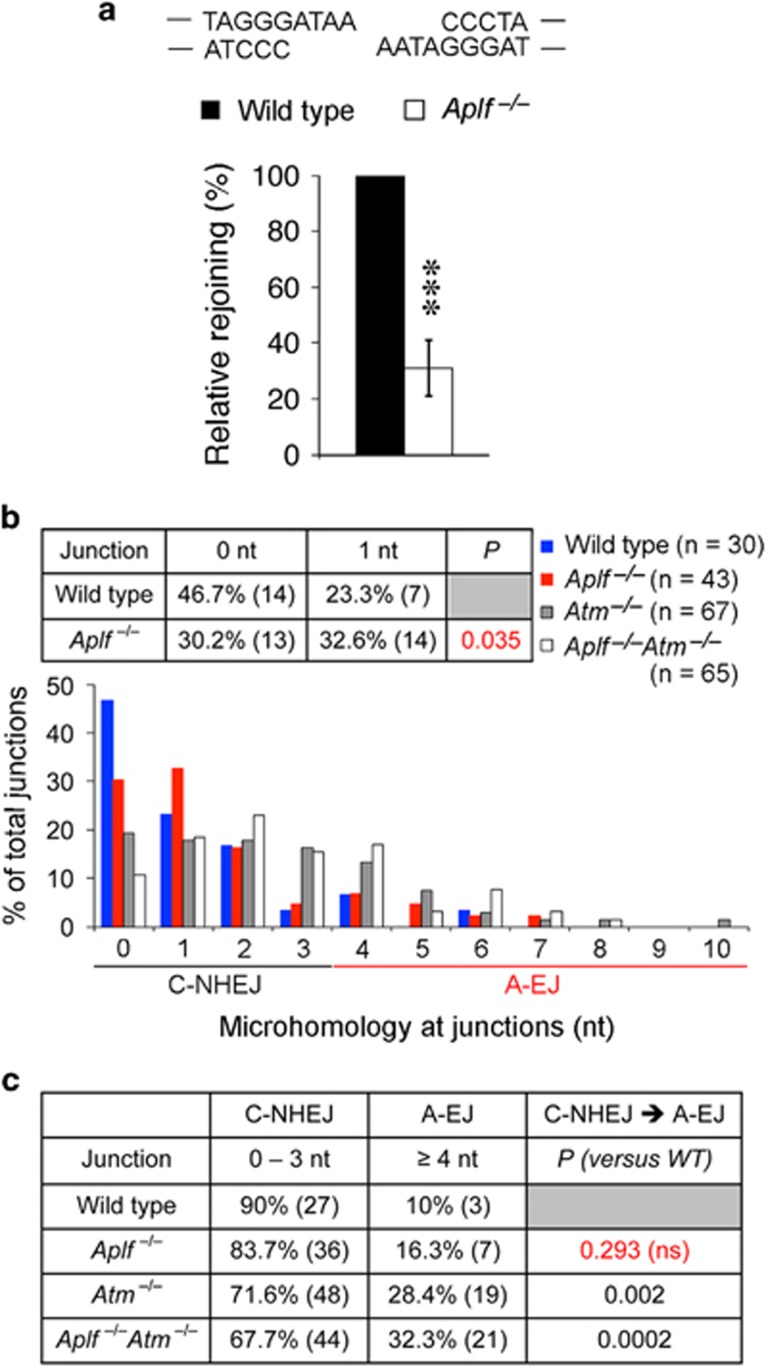
*Aplf*^–/–^ mice have lower extrachromosomal NHEJ and less DNA direct end joining at the switch junctions in B cells, but no switch to the alternative NHEJ (A-EJ) pathway. (**a**) Extrachromosomal NHEJ assays of I-SceI digested incompatible DNA ends. Data are means±standard deviation (S.D.) (*n*=6) relative to wild-type controls. ***P<0.001; unpaired two-tailed Student's *t*-test. (**b**) Microhomology at S*μ*–S*γ*1 junctions. The number of clones (*n*) sequenced was compiled from five independent experiments. nt, number of nucleotides at switch junctions. *P*<0.05 is considered statistically significant; two-tailed Fisher's exact test. (**c**) Frequencies of C-NHEJ (0–3 nt) and A-EJ (≥4 nt) at the S*μ*–S*γ*1 switch junctions. The *P*-values were evaluated by two-tailed Fisher's exact test against wild-type controls. NS, not significant

To assess the *in vivo* contribution of the NHEJ pathway in therapy-induced malignancies, we used a mouse model with reduced C-NHEJ activity by creating a frameshift disruption of the entire coding region of APLF (Supplementary Figure S1), in contrast to an earlier published APLF gene-trapped mouse model,^10^ whereby only the C-terminal half of the APLF was deleted leaving the binding sites for XRCC1/4 and Ku80 intact.^9, 12^ Although the C-NHEJ pathway functions in V(D)J recombination for B- and T-cell development^13^ and in class switch recombination (CSR)^14^ of immunoglobulin heavy chain (*IgH*), *Aplf*^–/–^ mice exhibited normal B- and T-cell development with mild reduction in the total count of splenocytes (Supplementary Figures S2A–C) and efficient CSR (Supplementary Figures S2D and E), suggesting that APLF is either not functional or functionally redundant in the immune system. In contrast, mouse embryonic fibroblasts (MEFs) from *Aplf*^–/–^ mice showed reduced NHEJ activity (Figure 1a) consistent with previous studies in human cell lines with depletion of APLF expression.^9^ On the other hand, *Aplf*^–/–^ mice displayed a reduction, albeit not elimination,^7^ of direct joins in the CSR switch junctions (Figure 1b and Supplementary Figure S3).^10^ Importantly, there was a concurrent increase of very short microhomology of one nucleotide at switch junctions, suggesting a change in the repairing pattern within the C-NHEJ pathway. Nevertheless, there was no significant increased use of longer microhomology (≥4 nt) and, therefore, we did not observe a switch from the C-NHEJ to the A-EJ pathway in *Aplf*^–/–^ mice (Figures 1b and c and Supplementary Figure S3). Additional depletion of APLF in mice with *Atm* null background (*Aplf*^*–/–*^*Atm*^–/–^) further decreased the number of direct joins compared with *Atm*^–/–^ mice reinforcing the notion that APLF is one of the factors in the C-NHEJ pathway (Figures 1b and c and Supplementary Figure S3). Together with the normal CSR activity in *Aplf*^–/–^ mice (Supplementary Figures S2D and E), these data strongly suggest that there is a moderate attenuation of C-NHEJ activity in these *Aplf*^–/–^ mice but the C-NHEJ pathway is functional and still predominating without switching to the A-EJ pathway. Loss of APLF may be compensated partially by other C-NHEJ factors to support C-NHEJ activity and to prevent a switch to the A-EJ pathway. As a major loss of C-NHEJ function, such as the deficiency of DNA-PKcs, Ku70, Ku80, XRCC4, DNA ligase 4, or Artemis, can lead to a number of intrinsic health issues such as growth defects, immunodeficiency, or inherent chromosomal instabilities,^13, 15^ the *Aplf*^–/–^ mouse, with moderate impairment of the C-NHEJ pathway and specific sensitivities to genotoxic treatments, becomes a good model to address therapy-induced malignant evolution.

### *Aplf*^–/–^ mice have higher p53-mediated cell death upon IR, but less dividing cells with DNA damage and lower frequency of translocation

**Figure 2:**
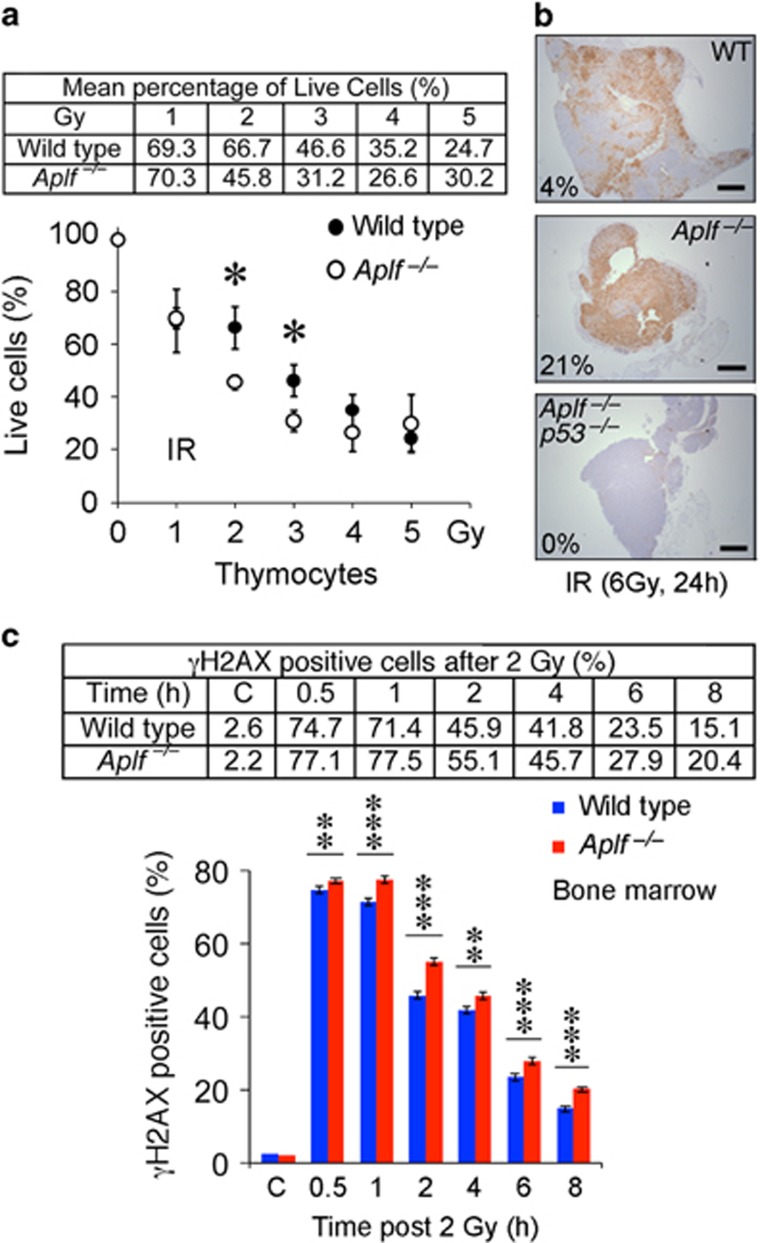
*Aplf*^–/–^ mice have higher p53-mediated cell death and higher *γ*H2AX-positive damaged cells. (**a**) Percentage of live thymocytes 24h after ionizing radiation (IR) (mean±S.D., normalized by non-irradiated controls; *n*=3). Gy, Gray. *P<0.05; unpaired two-tailed Student's *t*-test. (**b**) TUNEL staining of thymi from mice 24 h after whole-body 6 Gy IR. Percentage of area that stained TUNEL (apoptosis) positive, as depicted, was quantified by ImageJ software.^37^ Scale bars, 500 *μ*M. (**c**) Quantification of isolated bone marrow cells with ATM-mediated *γ*H2AX (pS139) DNA damage signal at the indicated time after 2 Gy IR (mean±S.D.; *n*=3). C, non-irradiated control. *P <0.05; **P<0.01; ***P<0.001; unpaired two-tailed Student's *t*-test

**Figure 3:**
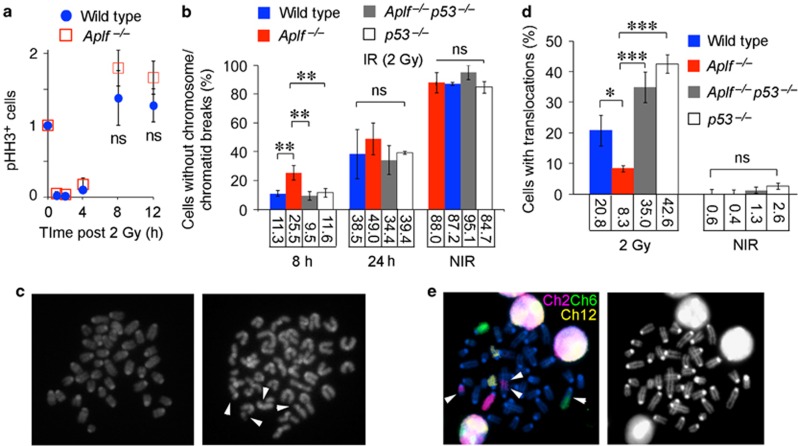
Wild-type and *Aplf*^–/–^ mice recovered similarly from IR-induced G2/M arrest but *Aplf*^–/–^ mice have less dividing cells with DNA damage and less IR-induced chromosomal translocation in the bone marrow. (**a**) The extent of the mitotic (M) phase was measured as the amount of phosphorylated histone H3 (pHH3, Ser10)-positive cells by flow cytometry and normalized by untreated controls. Data shown are mean±S.D. of four mice per group. NS, not significant. (**b**) Percentage of undamaged metaphases of non-irradiated (NIR) and irradiated (2 Gy) bone marrow cells 8 or 24h after IR. At least 50 metaphases from each mouse were randomly picked and scored (mean±S.D.; *n*=3 mice per group). *P<0.05; **P<0.01; ***P<0.001; one-way ANOVA with Tukey's *post hoc* test. NS, not significant. (**c**) Example of undamaged cell (left) and cell with DSBs (arrowhead, right) (× 630 magnification). (**d**) Percentage of bone marrow metaphases carrying chromosomal translocations 24h after 2 Gy IR. At least 50 metaphases from each mouse were randomly picked and scored (mean±S.D.; *n*=3 mice per group) *P<0.05; **P<0.01; ***P<0.001; one-way ANOVA with Tukey's *post hoc* test. NS, not significant. (**e**) An example of a cell with translocations (arrowheads, left) and DAPI stain of the same metaphase (right), chromosomes 2 (red), 6 (green), and 12 (yellow) (× 630 magnification)

Following IR, isolated thymocytes from *Aplf*^–/–^ mice in culture medium exhibited increased cell death after exposure to 2 and 3 Gray (Gy) (Figure 2a). Thymus of *Aplf*^–/–^ mice exposed to whole-body irradiation at 6 Gy also showed elevated p53-dependent apoptosis (4.1% in WT, 21.2% in *Aplf*^–/–^, 0% in *Aplf*^*–/–*^*p53*^–/–^) (Figure 2b). Following IR treatment, *γ*H2AX can be induced by ATM-mediated DDR^16, 17^ and by DNA-PK during apoptotic DNA fragmentation.^18^ In line with this, isolated bone marrow cells of *Aplf*^–/–^ mice in culture medium had higher *γ*H2AX-positive cells hours following exposure to 2 Gy IR (Figure 2c and Supplementary Figures S4), IR induced elevated level of stabilized p53 (Supplementary Figure S11), and IR enhanced p53 downstream target genes (Supplementary Figures S9A and B), indicating persistent DNA damage and likely higher apoptosis in the mutant mice. Since IR-induced DDR can elicit different outcomes including cell cycle arrest, DNA repair, apoptosis, and senescence,^16, 17^ not all cells with DNA damage and DSBs will pass onto the next generation of cell cycle. Therefore, we also look into the magnitude of DSBs in dividing cells. By 8 h post IR, both wild type and *Aplf*^–/–^ cells have already been released from IR-induced G2/M arrest and started to cycle normally (Figure 3a and Supplementary Figure S5). We found more bone marrow metaphases without chromosome or chromatid breaks in *Aplf*^–/–^ than wild type 8 h post IR (11.3% in WT *versus* 25.5% in *Aplf*^–/–^) (Figures 3b and c). However, this phenotype was reverted by the simultaneous deletion of *p53* (9.5% in *Aplf*^*–/–*^*p53*^–/–^) demonstrating that p53-mediated cell death could efficiently eliminate damaged cells from transmitting DNA damage to the daughter cells (Figure 3b). By 24 h following IR, the proportion of undamaged cycling cells was comparable in all four genotypes (wild type, *Aplf*^–/–^, *Aplf*^*–/–*^*p53*^–/–^, *p53*^–/–^) likely due to either DNA repair or clearance by cell death (Figure 3b). Since DNA damage can be repaired faithfully or unfaithfully, we therefore evaluated the extent of chromosomal aberrations after the induction of a large amount of DSBs by IR. Early evasion of damaged cells in *Aplf*^–/–^ mice by the p53-mediated cell death pathway led to a lower translocation incidence than in wild type (20.8% in WT *versus* 8.3% in *Aplf*^–/–^) (Figures 3d and e). Indeed, bone marrow metaphases from *Aplf*^*–/–*^*p53*^–/–^ and *p53*^–/–^ mice, in which the p53-dependent cell death function was impaired, had higher IR-induced chromosomal translocations (35% in *Aplf*^*–/–*^*p53*^–/–^ and 42.6% in *p53*^–/–^) than metaphases from either wild type or *Aplf*^–/–^ mice. These data demonstrate the important role of p53 in the reduction of IR-induced translocation in *Aplf*^*–/–*^ cells following irradiation.

### Depletion of APLF also reduces radiation-associated chromosomal aberration in human non-tumorigenic MCF10A cells

**Figure 4:**
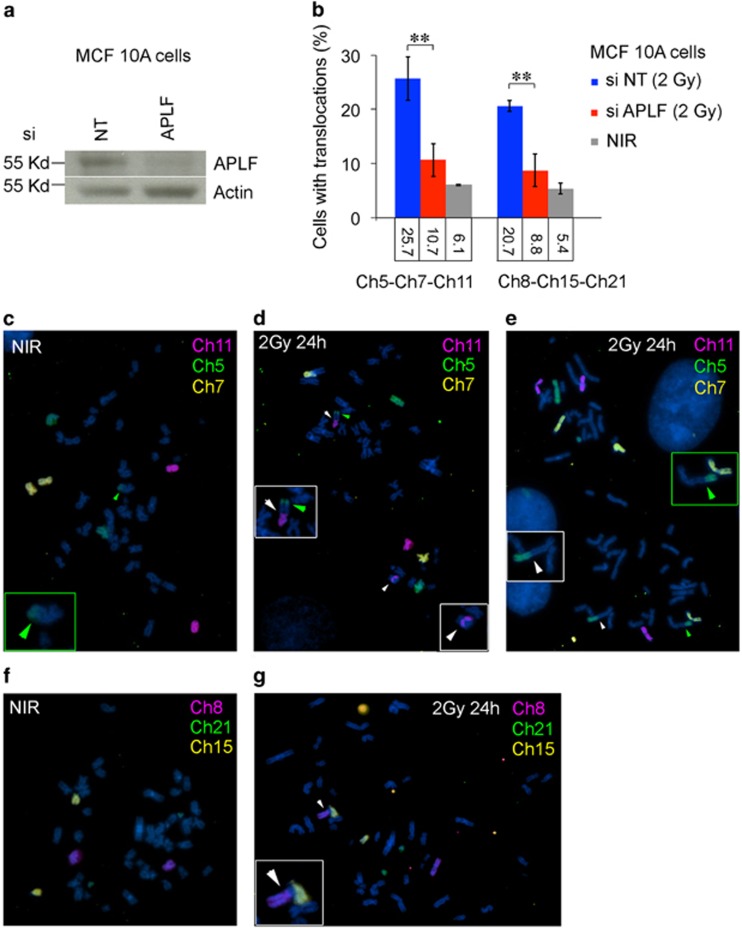
siRNA knockdown of APLF also reduced translocations in irradiated human non-tumorigenic MCF10A cell line. (**a**) Western blot showing the APLF protein level after 48-h treatment with a mixture of four siRNAs (Dharmacon) against *APLF* or a mixture of non-targeting negative siRNA control pool, which have been designed and microarray tested for minimal targeting of human, mouse, or rat genes (NT). (**b**) MCF10A cells were treated as described in (**a**) for 48 h before exposure to 2 Gy IR. Percentage of metaphases carrying chromosomal translocations of chromosomes 5, 7, or 11 (Ch5–Ch7–Ch11) and chromosomes 8, 15, or 21 (Ch8–Ch15–Ch21) was scored 24 h after irradiation. Fifty metaphases of each independent experiment were randomly picked and scored (mean±S.D.; *n*=3 independent experiments for each treatment type) **P<0.01. (**c**–**e**) Photomicrographs showing examples of chromosomal translocations and abnormalities in non-irradiated control (NIR, **c**) and irradiated (2 Gy; **d** and **e**) MCF10A cells for chromosomes 11 (red), 5 (green), and 7 (yellow). Green arrowhead depicts a background translocation that involves chromosome 5 found in MCF10A cell line in (**c**–**e**). This background translocation was not scored as IR-induced aberration (white arrowheads) and was excluded when evaluating translocation frequency described in (**b**). Insets are expanded views of the abnormal chromosomes found in each metaphase displayed. DNA was stained by DAPI, blue. (**f** and **g**) Photomicrographs showing examples of chromosomal translocations and abnormalities in non-irradiated control (NIR, **f**) and irradiated (2 Gy, **g**) MCF10A cells for chromosomes 8 (red), 21 (green), and 15 (yellow)

We attempted to examine if loss of APLF would similarly reduce the risk of chromosomal abnormalities in human cells. We knocked down APLF (APLF-KD) in the non-tumorigenic MCF10A human breast cell line by siRNA (Figure 4a). One day after exposure to 2 Gy, APLF-KD MCF10A cells had significant attenuation of chromosomal translocation and increased apoptosis compared with the non-targeting siRNA controls (Figures 4b–g and Supplementary Figures S9C and D). Therefore, like *Aplf*^–/–^ mice, depletion of APLF in human cells lowered the risk of IR-induced chromosomal abnormalities.

### IR-treated *Aplf*^–/–^ mice have less clonal chromosomal translocations in bone marrow 5 months after IR

**Figure 5:**
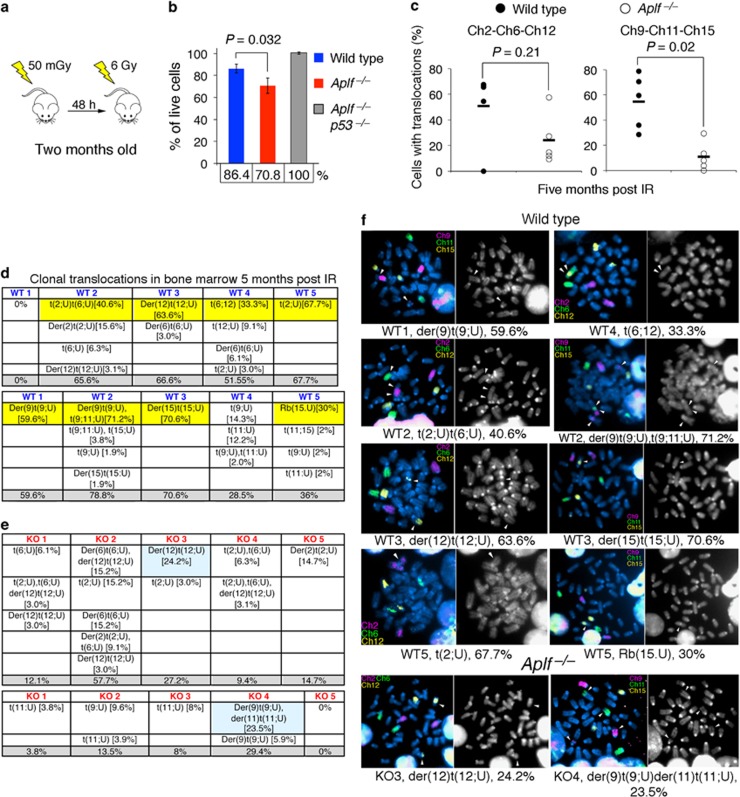
IR-treated *Aplf*^–/–^ mice have less clonal chromosomal translocations in bone marrow 5 months after IR. (**a**) Irradiation strategy. (**b**) Percentage of live thymocytes 24 h after whole-body *γ*-irradiation treated as described in (**a**) (mean±S.D., normalized by untreated controls; *n*=3). The *P-*values were derived from unpaired two-tailed Student's *t*-test. (**c**) Mice were irradiated as described in (**a**), and bone marrow cells were isolated five months post IR. Percentage of bone marrow cells with translocations involving chromosomes 2, 6, 12 (left) or chromosomes 9, 11, 15 (right). Each circle (open or closed) represents the frequency of cell with translocations by scoring at least 30 metaphases per mouse. Horizontal bar is the mean of five mice. The *P*-values were derived from two-tailed Mann–Whitney test. (**d**) The distribution of clonal translocations involving chromosomes 2, 6, 12 (top) or chromosomes 9, 11, 15 (bottom) in the irradiated wild-type mice as described in (**c**). Total percentage of translocation for each three-color chromosome panel per mouse is depicted at the bottom. t, translocation; (), type of chromosomal translocations; [ ], percentage of the described translocation; Der, derivative; Rb, Robertsonian translocation; U, chromosome other than chromosomes 2, 6, 12 for top panel or other than chromosomes 9, 11, 15 for bottom panel. (**e**) The distribution of clonal translocations in irradiated *Aplf*^–/–^ mice as described in (**c** and **d**). (**f**) Photomicrographs depict representative prominent clonal translocations in the bone marrow 5 months after IR as described in (**c**–**e**). Percentage of each clone is as indicated in parenthesis and only images of clones that accumulate 25% and above are shown. White arrowheads indicate the breakpoints in the rearranged chromosomes

We exposed animals to a small dose of whole-body irradiation at 50 milliGray (mGy) followed by a higher dose of 6 Gy after a 48-h interval to reduce radiation toxicity to normal tissues (Figure 5a).^19, 20^ *Aplf*^–/–^ mice invariably showed overall higher rates of p53-mediated cell death compared with wild-type mice (86.4% live cells in WT, 70.8% live cells in *Aplf*^–/–^), while the simultaneous disruption of *p53* in *Aplf*^–/–^ mice (*Aplf*^*–/–*^*p53*^–/–^) rescued the IR-induced cell death phenotype (Figure 5b). Not all cells with chromosomal translocations will survive and propagate over time. Cells with clonal translocations, which are able to expand, have likely acquired survival and proliferative advantages. Therefore, we also looked into the long-term effects of IR on the animals. Five months after radiation exposure, wild-type mice had two- to fivefold higher mean chromosomal translocation rates than those of the *Aplf*^–/–^ mice (Figure 5c). Most samples showed complex karyotypes. In particular, irradiated wild-type mice had one or more prominent clonal chromosomal rearrangements ranged from 30 to 70% in frequency, while those in *Aplf*^–/–^ mice were fewer with a frequency of 25% or lower (Figures 5d–f).

### Reduced C-NHEJ capacity in *Aplf*^–/–^ mice delays IR-induced dysplasia in hematopoietic cells and malignancy-related mortality

**Figure 6:**
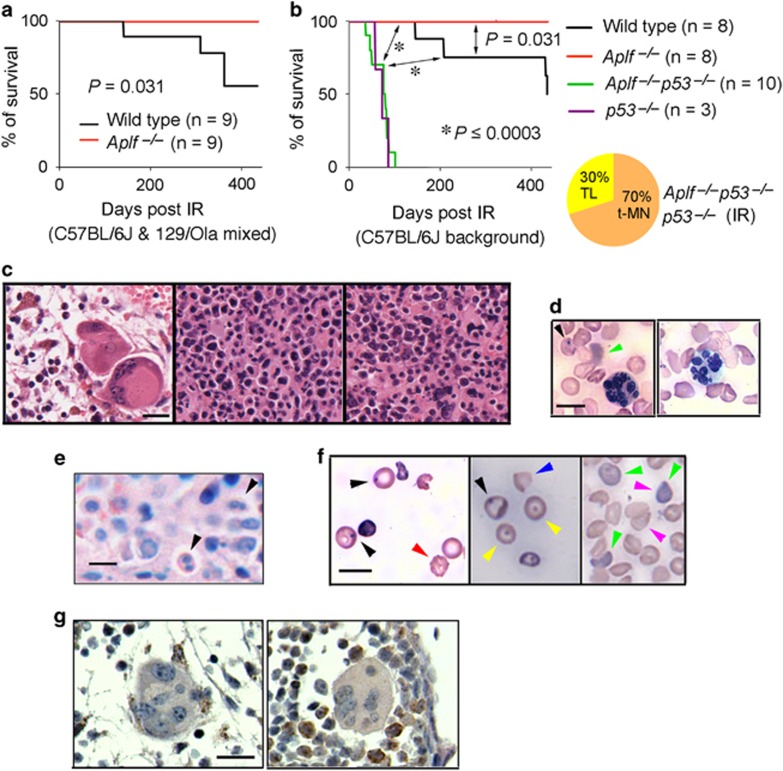
APLF deficiency delays IR-induced dysplasia in hematopoietic cells and malignancy-related mortality. (**a**) The Kaplan–Meier survival curve for mice of C57BL/6J and 129/Ola mix background irradiated as described in Figure 5a. The *P*-value was derived from log-rank test. (**b**) The Kaplan–Meier survival curve for mice of C57BL/6J background irradiated as described in Figure 5a. *P*-values were derived from log-rank test. TL, thymic lymphoma; t-MN, therapy-related myeloid neoplasm. (**c**) Bone sections of irradiated sick mice stained by hematoxylin and eosin (H&E). The left panel depicts a cluster of dysplastic megakaryocytes in the hypocellular bone marrow. Middle and right panels depict the hypercellular bone marrow. Scale bar, 20 *μ*M. (**d**) Dysplastic hypersegmented neutrophils (granulocytes) in the peripheral blood (Wright's stain) of irradiated moribund mice were observed. Black arrowhead depicts Howell-Jolly body inclusion in erythrocyte and green arrowhead depicts a polychromatic erythrocyte. Scale bar, 10 *μ*M. (**e**) The hypercellular bone marrow (Giemsa) displaying dysplastic binucleated erythroblasts (arrowheads). Scale bar, 10 *μ*M. (**f**) Peripheral blood (Wright's stain) of irradiated sick mice showing abnormal erythrocytes with Howell-Jolly bodies (black arrowhead), echinocyte (red arrowhead), target cells (yellow arrowhead), shistocyte (blue arrowhead), polychromatic erythrocytes (green arrowhead) and teardrop erythrocytes (magenta arrowhead), indicating dyserythropoiesis and dysfunctional bone marrow or spleens. Scale bar, 10 *μ*M. (**g**) Dysplastic megakaryocytes with multiple separate nuclear lobes were observed in irradiated sick mice with the hypocellular (left) or hypercellular (right) bone marrow stained by antimyeloperoxidase and hematoxylin. Scale bar, 20 *μ*M

To examine the impact of reduced NHEJ activity on therapy-related carcinogenesis, we monitored mouse cohorts exposed to 50 mGy followed by 6 Gy after 48 h. Irradiated wild-type mice began to develop IR-induced malignancy and died starting approximately 150 days after IR treatment (Figures 6a and b and Supplementary Figures S6 and S7). Conversely, *Aplf*^–/–^ mice, which showed higher IR-induced cell death (Figures 2a, b and 5b, and Supplementary Figure S9A) and lower chromosomal translocations (Figure 3d), had no malignancy-induced mortality more than 400 days post IR exposure (Figures 6a and b and Supplementary Figures S6 and S7). The survival was significantly shortened by the simultaneous deficiency of p53 on an *Aplf* null background (*Aplf*^*–/–*^*p53*^–/–^) (Figure 6b and Supplementary Figures S7 and S8A), which demonstrated high translocation frequency after IR (Figure 3d). Myeloid neoplasms resembling myeloid dysplasia^21^ were observed in these moribund or deceased irradiated wild type, *Aplf*^*–/–*^*p53*^–/–^, and *p53*^–/–^ animals. Of note, apart from about 30% of the irradiated *Aplf*^*–/–*^*p53*^–/–^ and *p53*^–/–^ mice that died immediately from thymic lymphoma, reminiscent of *p53*^–/–^ mice (Figure 6b and Supplementary Figure S8B),^22^ there was no development of thymic lymphoma identified in the necropsies from the rest of the IR-treated wild type, *Aplf*^*–/–*^*p53*^–/–^, and *p53*^–/–^ mice examined. These affected wild type, *Aplf*^*–/–*^*p53*^–/–^, and *p53*^–/–^ mice had either hypo- or hyper-cellular bone marrow (Figure 6c). Excessive proliferation of myeloblasts was not observed. In contrast, these animals showed abnormal hematopoiesis in peripheral blood and bone marrow, including hypersegmentation in granulocytes; Howell-Jolly bodies, polychromasia, and poikilocytosis in erythrocytes; binucleated erythroblasts; and dysplastic megakaryocytes with hypolobated nuclei or multiple separate nuclear lobes (Figures 6d–g). These features are consistent with single- and multi-lineage dysplasia and anemic conditions of human MDS or t-MDS. A case of neutropenia was also noted.

### IR-treated moribund wild-type mice have oncogenic clonal chromosomal aberrations

**Figure 7:**
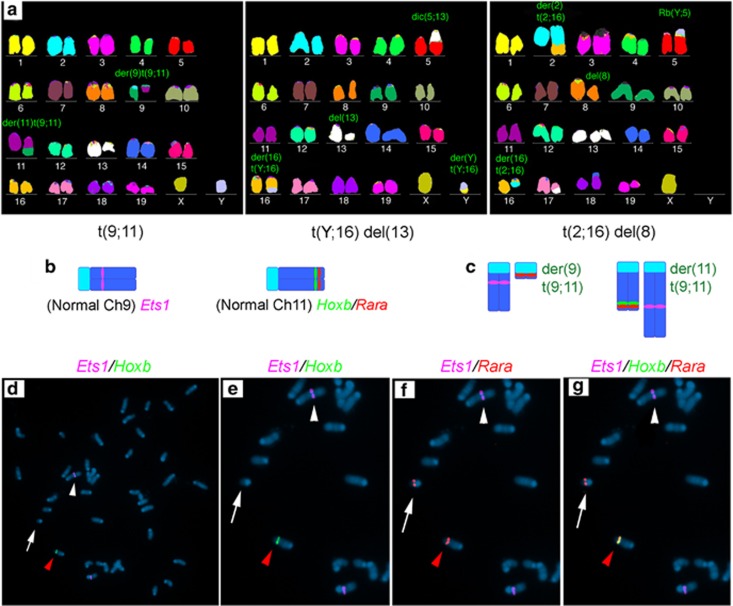
Oncogenic translocations and deletion found in irradiated moribund wild-type mouse. (**a**) M-FISH of spleen cells of a representative irradiated moribund wild-type mouse. Karyotypes of three clones of chromosomal abnormalities are shown (der, derivative; t, translocation; del, deletion; dic, dicentric chromosome; Rb, Robertsonian translocation). (**b**) Normal chromosomes 9 (Ch9) and 11 (Ch11) with indicated gene loci. (**c**) Cartoon for der(9)t(9;11) and der(11)t(9;11) rearranged chromosomes depicted in (**d**–**g**). (**d**–**g**) Two- and three-color FISH. The t(9;11) clonal translocation in (**a**, left) was verified by mouse BAC clones encompassing the locus of *Hoxb* gene cluster (green), *Ets1* (pink), or *Rara* (red). (**d**) Photomicrograph showing the entire metaphase of the t(9;11) clonal translocation where only one copy of the *Hoxb* gene cluster (green) was observed in the single normal chromosome 11 (red arrowhead). (**e**–**g**) Photomicrographs showing enlarged views of (**d**) with the normal chromosome 11 carrying the *Hoxb* gene cluster (green, red arrowhead), derivative chromosome 9 [der(9)t(9;11)] (white arrow), and derivative chromosome 11 [der(11)t(9,11)] (white arrowhead)

We examined an IR-treated moribund wild-type mouse cytogenetically and found complex chromosomal rearrangements of t(9;11), t(Y;16) del(13), and t(2;16) del(8) (Figure 7a). The t(9;11) clone, both in the spleen and the bone marrow (Figures 7b–g), showed a single copy deletion of the *Hoxb* gene cluster and translocations of both *Rara* and the oncogene *Ets1*. Downregulation of either *HOXB1*^23^ or *HOXB9*^24^ has been previously observed in a number of human AML cell lines and patient samples; hence, their functional roles in the proper differentiation of the myeloid lineage have been proposed. Chromosomal rearrangements of the *RARA*^25^ or the *ETS1*^26^ locus have also been reported in patients with myeloid neoplasms.

## Discussion

In this present study, we found that APLF deficiency in mice modestly reduced DNA damage repair and NHEJ activity. APLF-depleted cells, both *ex vivo* and *in vivo*, exhibited higher rates of radiation-induced p53-dependent cell death (Figures 2 and 5b) and fewer chromosomal translocations (Figures 3 and 5c–f) following IR exposure. IR-treated mutant mice showed a delay in malignancy-induced mortality. Simultaneous deficiency of p53 dampened IR-induced apoptosis and have reverted the benefit of impaired DNA repair on mortality in irradiated APLF-deficient mice. Depletion of APLF in non-tumorigenic human cells also markedly reduced the frequency of radiation-induced chromosomal aberrations.

Mice with deficiencies of both C-NHEJ (XRCC4 or DNA ligase 4) and p53 had increased incidence of B-cell lymphomas associated with *c-myc*/*IgH* oncogenic translocations. The *c-myc*/*IgH* breakpoint junctions of more than 50% of tumors studied were made of short homology (≤3 nt), typical of C-NHEJ.^27^ In wild-type mouse B cells, C-NHEJ has been found to promote *c-myc*/*IgH* rearrangements with blunt and low microhomolgy (1–3 nt) at breakpoints.^28^ Recent reports showed that extensive microhomology-mediated joining (≥4 nt) at rearrangement breakpoints, one of the major features of A-EJ, appears to be less frequent in *de novo* human translocations found in human cancer samples.^29, 30^ An analysis of primary human prostate cancers showed that most chromosomal fusions in these patient cancer tissues involved direct joins at the rearrangement junctions.^31^ Other studies in human breast and pancreatic cancers revealed that the most frequent junctions involved 0–3 nt at rearrangement breakpoints.^32, 33^ Our findings also showed higher rates of translocation in bone marrow cells of wild-type mice after exposure to IR, which was reduced by impairing C-NHEJ after knocking out *Aplf*. Depletion of APLF in the non-tumorigenic human breast cell line MCF10A also led to significant reduction of IR-induced chromosomal translocation. While genomic abnormalities in cancers are complex and diverse, the role of non-homologous end joining in somatic rearrangements is appreciated.^29, 30, 32^ The contribution of A-EJ to somatic translocations is still of particular interest since studies have showed an apparent interplay between C-NHEJ and A-EJ in modulating genome stability and translocations featuring longer microhomologies especially in cells lacking Ku or other core C-NHEJ factors. ^27, 34, 35, 36, 37^

Deletion of genetic sequences is found frequently in cells from human patients.^29, 30, 33^ We also detected both translocations and deletion of gene clusters after chromosomal rearrangement in irradiated wild-type mice and human non-tumorigenic MCF10A cells. Loss of part or all of chromosomes 5 (5q–/–5) and/or 7 (7q–/–7), complex cytogenetics, and a poor response to chemotherapy are characteristic findings in t-MN patients.^38^ Mutations found in t-MN patients include those observed in patients with *de novo* MDS and AML: *FLT3*, *cKIT*, *PTPN11*, *NRAS*, *RAS*, *BRAF*, *c-FMS*, *JAK2*, *CEBPA*, *TP53*, and *AML1* genes.^39^ Previous studies suggest that about 30% of t-MN patient carry loss-of-function mutations in *TP53*.^4, 38^ A recent study indicates that the high frequency of *TP53* mutations in t-MN patients may not be directly induced by genotoxic treatments. But, rather, when there are age-related loss-of-function mutations of *TP53* in rare hematopoietic stem/progenitor cells, these cells may become resistant to chemo- and radiotherapies and hence can preferentially expand even after treatment.^4^ Another rational explanation, as demonstrated by the present study, is that when the p53-mediated cell death pathway is impaired due to mutations in *TP53*, instead of clearing away severely damaged cells, more cells carrying DNA DSBs will survive and are repaired by the C-NHEJ pathway faithfully and unfaithfully. Therefore, mutations in *TP53* are frequent in t-MN patients and are associated with the higher risk of chromosomal abnormality formation and malignant transformation after genotoxic therapies.

Li *et al.* have generated mice bearing lysine to arginine mutations at the acetylation sites in p53. These mutations did not affect the metabolic regulation and antioxidant function of p53 but seemed sufficient for inhibiting early-onset spontaneous thymic lymphoma usually found in p53 null mice.^22, 40^ Cellular DNA damages trigger a spectrum of signaling which leads to the stabilization of p53, ATM/ATR-associated direct phosphorylation of Ser18 of p53 in mice (Ser15 in human p53) and ATM/ATR-associated indirect phosphorylation of Ser23 of p53 in mice (Ser20 in human p53) via chk2 protein kinase.^16, 17^ Mice that harbor alanine substitutions of these two key DNA damage-targeted phosphorylation sites of Ser18 and Ser23 in p53 are resistant to IR-induced p53-dependent apoptosis. These alanine substituted p53 mutant mice developed an array of malignancies, thereby reinforcing a link between p53-dependent DDRs and tumor suppression.^41^ Another study also showed that mice with a single alanine substitution at Ser18 of p53, instead of developing early-onset spontaneous tumors like those found in p53 null mice, were succumbed to late-onset lymphomas, demonstrating that DDR function of p53 is important to tumor suppression *in vivo*.^42^

The current study demonstrates that the C-NHEJ pathway plays a role in producing genomic abnormalities in particular following the induction of a large number of DSBs from genotoxic treatments. We conclude that the more error-prone NHEJ DNA damage repair, even when the classical NHEJ pathway predominates, can promote genomic instability in normal tissues after genotoxic therapies. Carcinogenesis can be driven by complex chromosomal rearrangements, deletions or mutations. Manipulation of the efficiency in DNA damage repair may have the potential to intervene in the development of these diseases.

Genotoxic therapy-induced cell death is the common rationale behind most cancer therapies. This approach needs to be balanced against the risk of therapy-induced acute and late toxic side effects in normal tissues, such as anemia, fibrosis, and second cancers, which may limit the efficacy of therapy that can be administered to patients.^43^ This is an important area of investigation that warrants further exploration in future studies. Central to this notion is whether sensitizing *TP53* during cancer therapies augments therapy-related toxicity. The present study demonstrates that one of the most serious and deadly therapy-induced toxic side effects, second cancers, could potentially be reduced. Indeed, the balance of excessive cell death and evasion of damaged cells need to be carefully controlled to maximize the benefits of *TP53* as the guardian of genome.

## Materials and Methods

### Antibodies and reagents

Anti-mouse B220 (Clone RA3-6B2), CD43 (Clone eBioR2/60), IgM (Clone II/41), CD3 (Clone 145-2C11), CD4 (Clone RM4-5), CD8 (Clone 53-6.7) and 7-amino-actinomycin D (7-AAD) were obtained from eBioscience (San Diego, CA, USA). Anti-mouse IgG1 (Clone A85-1), *αβ*TCR, Streptavidin Particles Plus-DM, and Annexin V-FITC were from BD Biosciences (San Jose, CA, USA). APLF antibody (N-16) and actin antibody (C-11) were from Santa Cruz (Dallas, TX, USA). Recombinant murine interleukin 4 (IL-4), IL-3, IL-6, Flt3-ligand, stem cell factor (SCF), TPO, and recombinant human TSLP were from Peprotech (Rocky Hill, NJ, USA), lipopolysaccharides (LPS) from Sigma (Oakville, ON, Canada), propidium iodide (PI) from Invitrogen (Waltham, MA, USA), and red blood cell lysing buffer from Sigma.

### Gene-targeted deletion of murine *Aplf* locus

The targeting construct was designed to replace exons 2–3 of *Aplf* by *PGK* promoter-driven neomycin resistance gene, which would create frameshift mutation starting within the Forkhead associated domain. Targeting vector was linearized by *Not*I digestion and electroporated into E14K embryonic stem (ES) cells (129/Ola). G418-resistant ES cell clones were then screened for homologous recombination by nested PCR. Candidate ES cells clones were further confirmed by Southern blot with PCR-generated probes (1 and 2) as described in Supplementary Figure S1A, using the standard method. Four correctly targeted clones were confirmed and three were injected into C57BL/6J blastocysts. Two independent mouse lines were successfully established using standard procedures.

### Southern blot analysis

Genomic DNA was extracted from the indicated ES cell clones. *Bam*HI-digested DNA was resolved by electrophoresis, transferred onto nitrocellulose membrane, and hybridized by ^32^P radioactive probes using the standard method as described in the legend of Supplementary Figure S1A. Probed membranes were exposed to storage phosphor screen (Perkin Elmer, Waltham, MA, USA). Images were captured by the Typhoon Trio Imager (GE Healthcare, Chicago, IL, USA).

### Primers

*Aplf*^*–/–*^ mice were genotyped using following primers: (2637U) 5′-CTTACTGGGCTTGACTTTAGAC-3′ (3570) 5′-GGCTTGATTCGAAGCTGACTAT-3′ and (2995L) 5′-CATCGCCTTCTATCGCCTTCTT-3′ as primers a, b, c described in Extended Data Figure S1a. The following primers were used for the semiquantitative PCR: *Aplf*, forward 5′-GAATCAAGCCAATCCATAGGAATC-3′ and reverse 5′-CATCTGACTGTTTCTCAGAGTAC-3′. The following primers were used for qRT-PCR: *Puma* forward 5′-CTGGAGGGTCATGTACAATCTCTT-3′ and reverse 5′-CACCTAGTTGGGCTCCATTTCT-3′ *mdm2*, forward 5′-GGATCTTGACGATGGCGTAAG-3′ and reverse 5′-AGGCTGTAATCTTCCGAGTCC-3′ *APLF1*, forward 5′-CCGCTGCTGGGAATAACAGA-3′ and reverse 5′-GCTTCAATGGTAAGAGCTGACT-3′ *APLF2*, forward 5′-CAGCTCTTACCATTGAAGCCA-3′ and reverse 5′-CCAGTTGTGAGGCACCAGTAG-3′ primers.

### Animal studies

Procedures for maintaining mice and all experiments described were approved by the Ontario Cancer Institute Animal Facility and were performed in compliance with the regulations of the Animal Ethics and Animal Care Committees at the Princess Margaret Cancer Centre. Unless indicated in the figure legends, *Aplf*^*–/–*^ mice used in the analyses have been backcrossed with C57BL/6J mice for six generations. *p53*^*+/–*^ mouse was purchased from Taconic. *Atm*-knockout mouse^44^ was generated by Peter J McKinnon (St. Jude Children's Research Hospital, Memphis, TN, USA) and kindly provided by Tak W Mak (Ontario Cancer Institute, Toronto, ON, Canada). The backcrossed *Aplf*^*–/–*^ mice were used to generate *Aplf*^*–/–*^*Atm*^*–/–*^ and *Aplf*^*–/–*^*p53*^*–/–*^ mice by mating *Aplf*^*–/–*^ mice with *Atm*^*+/–*^ or *p53*^*+/–*^, respectively. For mouse cohort studies, mice of 2 months old were exposed firstly to 50 mGy (at 28.84 cGy/min) of irradiation, followed by 6 Gy (at 108.25 cGy/min) treatment 48 h later by X-RAD 320, an X-ray irradiator (Precision X-Ray, North Branford, CT, USA). Mice were killed and necropsied at end points of either morbidity or mortality. Mouse tissues were extracted and processed as described in the Histology section.

### Lymphocyte development, class switch recombination, and switch junction analyses

For analysis of lymphocyte development, single-cell suspensions were prepared from the bone marrow, thymus, and spleen from mice of 6–8 weeks old. 1 × 10^6^ cells were stained by fluorescence-conjugated antibodies as indicated. Data were obtained by FACSCalibur flow cytometer (BD Biosciences) and interpreted by FLOWJO software (Tree Star, Ashland, OR, USA). For class switch recombination assays, splenic CD43^–^ B cells were isolated by immunomagnetic depletion using biotinylated anti-CD43 and streptavidin particles. Cells at 5 × 10^5^/ml were stimulated for 4 days by 10 ng/ml of IL-4 plus 20 *μ*g/ml of LPS in RPMI-1640-rich medium (containing 2 mM l-glutamine, 10 mM HEPES, 1 mM sodium pyruvate, 4.5 g/l glucose, and 1.5 g/l sodium bicarbonate) supplemented with 10% (vol/vol) fetal bovine serum (FBS) and 50 *μ*M *β*-mercaptoethanol. Data were obtained by flow cytometry as described above. For S*μ*–S*γ*1 switch junction analysis, splenic CD43^–^ B cells were isolated and stimulated by IL-4 and LPS for 4 days as mentioned above. The S*μ*–S*γ*1 junction regions were amplified by PCR from extracted genomic DNA and cloned into PCR2.1 vector for sequencing using Topo-TA cloning kit (Invitrogen) as described previously.^45^ Sequence data were analyzed by pairwise sequence alignment tool (EMBOSS, EMBL-EBI) using GenBank files MUSIGCD07 (for S*μ*) and MUSIGHANB (for S*γ*1) for IgM and IgG1 switch regions, respectively.

### Cell death assay

Isolated thymocytes were maintained *ex vivo* in RPMI-1640-rich medium supplemented with 10% FBS and 50 *μ*M *β*-mercaptoethanol. Cells were either treated or non-treated by *γ*-irradiation using Cs137 based Gammacell 40 Exactor (Nordion International Inc., Ottawa, ON, Canada) at 0.78 Gy/min to induce DNA damage with dosage as indicated. Twenty-four hours later, cells were co-stained by Annexin V-FITC and PI or 7-AAD, and analyzed by flow cytometry. Proportion of live cells was determined by estimating percentage of cells negative to both Annexin V and PI (or 7-AAD) staining. The percentage of live cells was normalized by non-irradiated controls. For whole-body *γ*-irradiation, 4- to 6-week-old mice were *γ*-irradiated as described in the text and figure legends. The thymus was extracted from mice 24 h after IR treatment, stained, and analyzed as mentioned above. MCF10 cells were transfected with control and *APLF* siRNA. After 48 h, the cells were treated by 2 Gy IR and apoptotic cells were quantified with Annexin V and PI staining.

### Histology

Thymus tissue was extracted from mice after *γ*-irradiation as described in the figure legends, fixed in neutral buffered formalin (Sigma), paraffin-embedded, sectioned, and stained by TUNEL. Buffered formalin fixed femurs were paraffin-embedded after decalcified with 10% EDTA, pH 7.4. Paraffin sections on slides were heated by microwave in 50 mM citrate buffer for antigen retrieval. Sections were stained by antimyeloperoxidase (ab9535; Abcam, Cambridge, MA, USA) using the standard methods. DNA was counterstained by hematoxylin. Hematoxylin and eosin (H&E) and Giemsa stainings of paraffin-embedded sections were prepared by standard procedures. Peripheral blood was collected by the cardiac puncture method and blood smears were stained by Wright's stain (EMD Millipore, Billerica, MA, USA) according to the supplier's protocol.

### Extrachromosomal NHEJ assay

Primary MEFs of wild type and *Aplf*^*–/–*^ mice were generated from mouse embryos at E13.5 and immortalized by SV40 T antigen using the standard method. Immortalized MEFs were maintained in DMEM high-glucose (4.5 g/l) medium supplemented with 10% FBS and 0.8 *μ*g/ml of puromycin. I-SceI-linearized *pEGFP-Pem1-Ad2*^46^ was co-transfected with *pDsRed-Mono-N1* (Clontech, Mountain View, CA, USA), as the transfection control, into immortalized MEFs by Amaxa Nucleofector MEF 1 reagent (Lonza, Basel, Switzerland) following the manufacturer's manual. Cells were analyzed 48 h post transfection by flow cytometry using the FACSCalibur and FLOWJO software to estimate re-circularized *pEGFP-Pem1-Ad2*. Rejoining efficiency was determined by the ratio of GFP^+^/RFP^+^ cells, relative to that of wild-type controls.

### *γ*H2AX-positive cells quantification

Total bone marrow cells were extracted from femurs and tibias of mice using phosphate-buffered saline (PBS) supplemented with 1% (vol/vol) FBS and 1 mM EDTA. After lysing red blood cells, 1 × 10^6^/ml of nucleated cells were cultured in RPMI-1640-rich medium supplemented with 10% FBS and 50 *μ*M *β*-mercaptoethanol, recombinant murine Flt3-ligand (20 ng/ml), IL-3 (20 ng/ml), IL-6 (20 ng/ml), SCF (20 ng/ml), TPO (50 ng/ml), and recombinant human TSLP (10 ng/ml) for 16 h. After exposed to 2 Gy IR, bone marrow cells were harvested at indicated time points, fixed in 2% paraformaldehyde (Sigma), and permeabilized by 90% methanol.^47^ Fixed cells were then stained by anti-*γ*H2AX (pS139) conjugated with Alexa Fluor 647 (BD Biosciences) at 4 °C for 1 h, followed by PI (30 *μ*g/ml) in the presence of 100 *μ*g/ml RNase A (Thermo Scientific, Waltham, MA, USA) for 60 min at room temperature in dark. At least 10 000 cells of each sample were analyzed as described^47^ by FACSCanto (BD Biosciences) and interpreted by FLOWJO software.

### Cell cycle analysis

Red blood cell-depleted spleen cells were cultured at 1 × 10^6^/ ml in RPMI-1640-rich medium supplemented with 10% FBS and 50 *μ*M *β*-mercaptoethanol, IL-4 (10 ng/ml), and LPS (20 *μ*g/ml) for 48 h. Cells were then irradiated (2 Gy, X-RAD 320), harvested at the indicated time as described in the text and figures, and fixed in ice-cold 70% ethanol. Fixed cells were stained by PI (27 *μ*g/ml) in the presence of 100 *μ*g/ml RNase A for 60 min at room temperature in dark. DNA content was measured by flow cytometry and analyzed by FLOWJO. To assess the G2/M checkpoint by flow cytometry, fixed cells were permeabilized in 0.1% Triton X-100/PBS buffer for 15 min at room temperature before staining by Alexa Fluor 488-conjugated phospho-histone-H3 (pSer10) antibody (New England BioLabs, Ipswich, MA, USA).

### *Ex vivo* bone marrow metaphase preparation

Total bone marrow cells were extracted from femurs and tibias of mice using PBS supplemented with 1% FBS and 1 mM EDTA. After lysing red blood cells, nucleated cells were cultured in RPMI-1640-rich medium supplemented with 10% FBS and 50 *μ*M *β*-mercaptoethanol, recombinant murine Flt3-ligand (20 ng/ml), IL-3 (20 ng/ml), IL-6 (20 ng/ml), SCF (20 ng/ml), TPO (50 ng/ml), and recombinant human TSLP (10 ng/ml). After 24 h, bone marrow cells were exposed to 2 Gy IR (X-RAD 320). One hour before harvesting cells, 150 ng/ml of colcemid (Roche, Basel, Switzerland) was added to the culture. Cells were then harvested, treated in 75 mM potassium chloride (KCl) at 37 °C for 20 min, and fixed with ice-cold methanol/acetic acid (3:1) fixative. Fixed cells were then dropped on slides to generate metaphase spreads. Slides were allowed to dry overnight and then mounted with Vectorshield containing DAPI (Vector Laboratories, Burlingame, CA, USA) for analysis. Images were taken with AxioImager Z1 (Zeiss, Oberkochen, Germany) equipped with Metamorph software (Molecular Devices, Sunnyvale, CA, USA).

### Direct bone marrow metaphase preparation

Total bone marrow cells from whole-body irradiated mice were extracted by RPMI-1640-rich medium supplemented with 10% FBS, 50 *μ*M *β*-mercaptoethanol, and 10 U heparin/ml (Sigma). Cells were treated in 75 mM KCl containing 40 ng/ml of colcemid at 37 °C for 20 min, fixed with ice-cold methanol/acetic acid, and dropped on slides. Air-dried slides were processed for two- and three-color FISH or three-color whole chromosome paint as described below.

### Multicolor FISH (M-FISH)

Red blood cell-depleted spleen cells were cultured in RPMI-1640-rich medium supplemented with 10% FBS and 50 *μ*M *β*-mercaptoethanol, recombinant murine Flt3-ligand (20 ng/ml), IL-3 (20 ng/ml), IL-6 (20 ng/ml), SCF (20 ng/ml), TPO (50 ng/ml), IL-4 (10 ng/ml), recombinant human TSLP (10 ng/ml), and LPS (20 *μ*g/ml) for 48 h. One hour before harvesting cells, 150 ng/ml of colcemid (Roche) was added to the culture. Cells were then harvested, treated in 75 mM potassium chloride (KCl) at 37 °C for 20 min, and fixed with ice-cold methanol/acetic acid (3:1) fixative. Fixed cells were then dropped on slides. Slides containing metaphases were stained with mouse m-FISH kit (21XMouse) to identify chromosome aberrations according to the supplier's protocol (MetaSystems, Altussheim, Germany). Metaphase spreads were captured at × 630 magnification on an Imager M1 Zeiss microscope (Carl Zeiss Canada Limited, Toronto, ON, Canada) equipped with the appropriate filters. The JAI CV-M4+CL progressive scan monochrome camera (JAI Inc., San Jose, CA, USA) and the MetaSystems Isis FISH Imaging software programs v5.3 (MetaSystems) were used for analysis. Gene-specific probes were applied, as described below, to confirm specific translocations as M-FISH has some limitations in delineations of chromosomal rearrangements.

### Mouse BAC clones probe preparation

Mouse BAC clones encompass the locus containing *Hoxb* (RP23-205E11 and RP23-9G13), *Ets1* (RP23-403F11 and RP23-101N3), *Sept4* (RP23-333D13), or *Rara* (RP23-333D2 and RP23-364P11). BAC clones were verified by PCR using primers against regions of each gene. Probes were prepared by nick translation kit using green dUTP, orange dUTP, or Aqua dUTP according to the manufacturer's protocol (Abbott Molecular, Des Plaines, IL, USA). At least 100 ng of each probe was used in hybridization.

### Two- and three-color FISH

Metaphases on slides were treated by 100 *μ*g/ml RNase A (Thermo Scientific) in 2 × SSC for 1 h at 37 °C, washed 2 × 5 min in 2 × SSC. Fixed in 4% formaldehyde for 10 min. Washed 2 × 5 min in 2 × SSC and dehydrated in 70, 90, and 100% ethanol. The slides were then treated with 70% formamide in 2 × SSC at 75 °C for 5 min. Rinsed briefly in cold 2 × SSC, once in 70% ethanol (5 min, –20 °C), and dehydrated with 90 and 100% ethanol at room temperature. Fluorescent probes were denatured in 50% Formamide, 10% (w/v) dextran sulfate, 1 × SSC and 20 × (w/w) mouse Cot1 DNA (Invitrogen) at 75 °C for 10 min, applied to the slides, and incubated overnight in a humidified chamber at 37 °C. Slides were then washed once in 0.4 × SSC/0.3% Tween 20 at 74 °C for 5 min and once in 4 × SSC/0.1% Tween 20 at room temperature for 2 min. Dehydrated, air dried, and mounted with Vectorshield containing DAPI. Images were obtained using AxioImager Z1 equipped with Metamorph software.

### Whole chromosome paint

Metaphases were stained by 3-color whole mouse chromosome paint (Applied Spectral Imaging, Carlsbad, CA, USA) in red (chromosome 2 or 9), green (chromosome 6 or 11), and aqua (chromosome 12 or 15) or by three-color whole human chromosome paint (Applied Spectral Imaging) in red (chromosome 8 or 11), green (chromosome 5 or 21), and aqua (chromosome 7 or 15) according to the manufacturer's protocol. Slides were counterstained with Vectorshield containing DAPI. Images were obtained using AxioImager Z1 equipped with Metamorph software.

### MCF10A cell culture

MCF10a cells were maintained in DMEM/F12 medium supplemented by 2 mM l-glutamine, 5% horse serum (Life Technologies), 20 ng/ml epidermal growth factor (Peprotech), 0.5 *μ*g/ml hydrocortisone (Sigma), 0.1 *μ*g/ml cholera toxin (Sigma), 10 *μ*g/ml insulin (Sigma), and 50 U/ml of penicillin–streptomycin.

### siRNA knockdown of APLF

All siRNA pools were obtained from Dharmacon. For every experiment, 25 nM of four siRNA sequences were used to transfect cells by Dharmafect 1 (Dharmacon, Lafayette, CO, USA) according to the manufacturer's protocol. Cells were treated by 2 Gy IR or harvested for western blot 48 h after.

siRNA sequences against: human *APLF*, GCACAAGAUAGAAUAUAGA, CUUCAUAUUACGUGACUUU, AGCAAUCAGUGGAGGUAAU, UGAUUAUGGAGGUGUACAA, and non-targeting control, UGGUUUACAUGUCGACUAA, UGGUUUACAUGUUGUGUGA, UGGUUUACAUGUUUUCUGA, UGGUUUACAUGUUUUCCUA.

### Statistical analysis

Differences between groups were assayed using an unpaired two-tailed Student's *t*-test after evaluation of variance. In cases where the assumption of the *t*-test was not valid, the non-parametric two-tailed Mann–Whitney test was used. Multiple groups were analyzed by one-way ANOVA with Tukey's *post hoc* test. A two-tailed Fisher's exact test was used to analyze the contingency table of microhomology length at switch junctions. Survival differences were analyzed by log-rank test. *P*<0.05 was considered statistically significant.

## Abbreviations

APLF: aprataxin and polynucleotide kinase/phosphatase (PNKP)-like factor
Xip1: XRCC1-interacting protein 1
C2orf13: chromosome 2 open reading frame 13
PALF: PNK and APTX-like FHA protein
IR: ionizing radiation
t-MNs: therapy-related myeloid neoplasms
t-MDS: therapy-related myelodysplastic syndrome
t-AML: therapy-related acute myeloid leukemia
ATM: ataxia telangiectasia mutated
ATR: ATM and RAD3-related
DSB: DNA double-strand break
DDR: DNA damage response
NHEJ: non-homologous end joining
C-NHEJ: classical NHEJ
A-EJ: alternative NHEJ
nt: nucleotides
PAR: poly(ADP-ribose)
PARP3: PAR polymerase 3
ROS: reactive oxygen species
XRCC4: X-ray repair cross-complementary protein 4
PNKP: polynuceotide kinase/phosphatase
CSR: class switch recombination
IgH: immunoglobulin heavy chain
MEFs: mouse embryonic fibroblasts
DNA-PKcs: DNA-dependent protein kinase catalytic subunit
Gy: Gray
mGy: milliGray
KD: knock down
WT: wild type
RARA: retinoic acid receptor alpha
FLT3: Fms-like tyrosine kinase 3
PTPN11: tyrosine-protein phosphatase non-receptor type 11
NRAS: neuroblastoma RAS viral oncogene homolog
LPS: lipopolysaccharides
PI: propidium iodide
ES: embryonic stem
CEBPA: CCAAT/enhancer-binding protein alpha

## Supplementary Material

Supplementary Informations
