# Supplementary material for: Attenuated DNA damage repair delays therapy-related myeloid neoplasms in a mouse model
Source: Cell Death Dis. 2016 Oct 6;7(10):e2401–. doi: 10.1038/cddis.2016.298 (PMC5133969; doi:10.1038/cddis.2016.298)
Supplement: Supplementary Informations [file cddis2016298x1.docx]

Supplementary Information

Figure S1


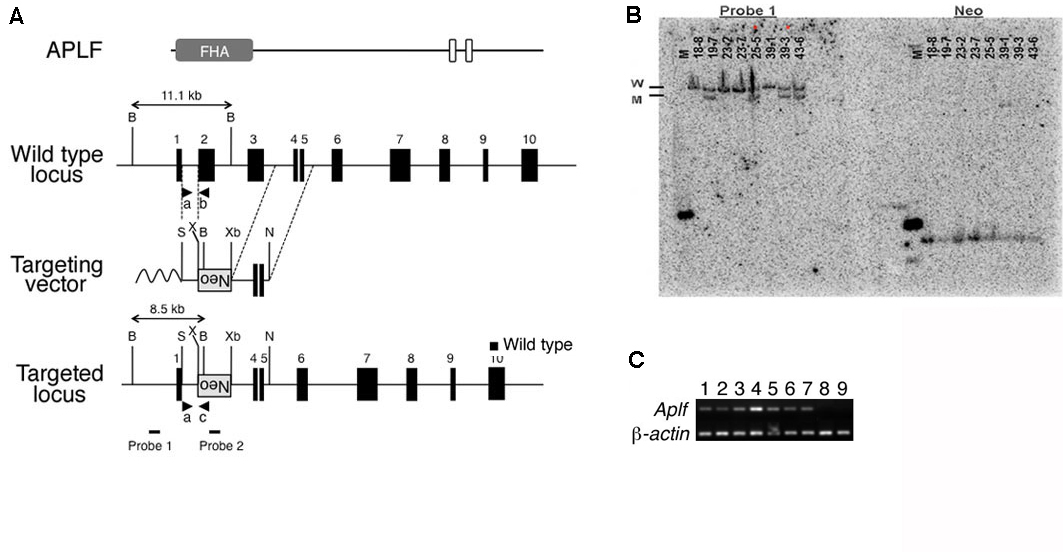


**Figure S2**

**
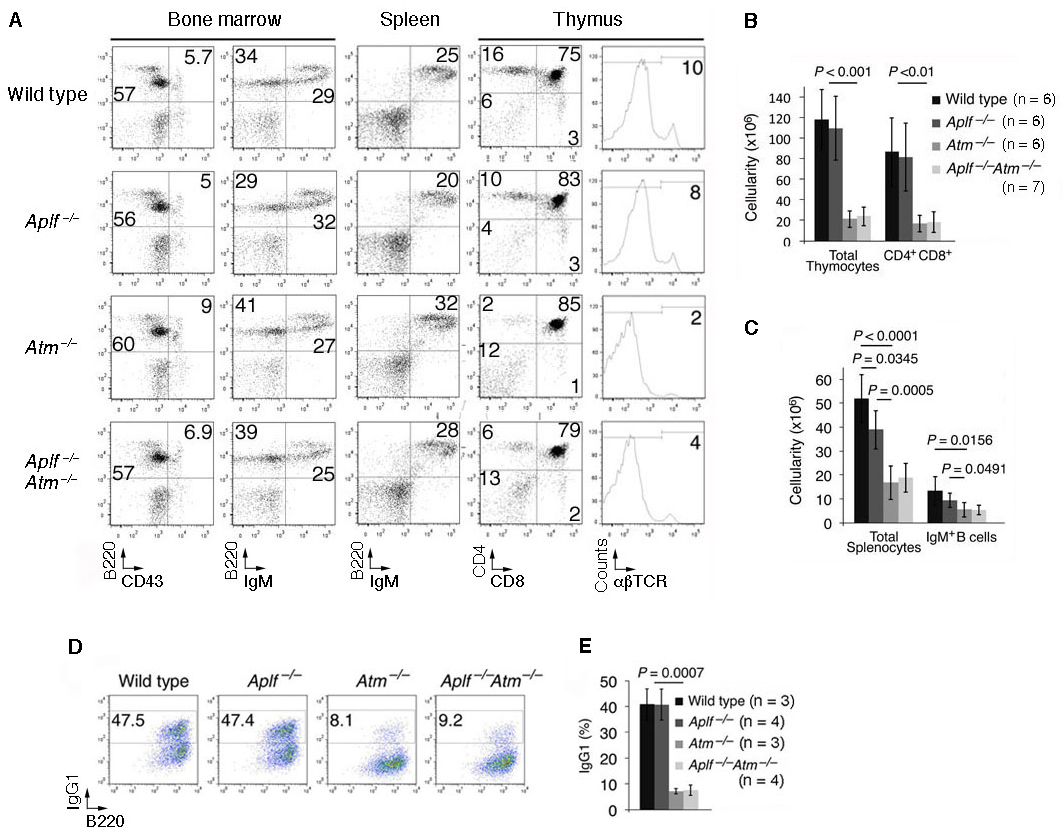
**

**Figure S3**

**Wild type**

S9wt-4

MUSIGCD07 (Sμ)

5140 gcttgagccaaaatgaagtagactgtaatgaactggaatgagctgggccgctaagctaaactaggctggcttaaccg **Dir**

GCTTGAGCCAAAATGAAGTAGACTGTAATGAACTGGAATGAATGAGAGCTGGAGCTAGTATGAAGGTGGAGGTCCAG

8972 gatcagggatagacatgtaagcagtcaagctcagctactacatgagagctggagctagtatgaaggtggaggtccag

MUSIGHANB (Sγ1)

S9wt-2

5319 Tcagctatgctacgc-gtgttggggtga**g**ctgatctgaaatgagctactctggagtagctgagatggggtgagatgg **Dir**

TCAGCTATGCTACGCTGTGTTGGGGTGA**C**CTGATCTGTGAAGGTAA**A**CTGGAGCTAGTGGGGGTGTGGGAGACCAGGC

8879 ggagctgatgggtgtataaggtaccaggctgagcagctgaaggtaa**c**ctggagctagtgggggtgtgggagaccaggc

S11wt-3

5213 accgagatgagccaaactggaatgaacttcattaatctaggttgaatagagctaaactctactgcctacactggacT **Dir**

ACCGAGATGAGCCAAACTGGAATGAACTTCATTAATCTGCTGGAGCTAGTATGAAGGTGGAGGTCCAGTTGAGTGTC

8981 tagacatgtaagcagtcaagctcagctactacatgagagctggagctagtatgaaggtggaggtccagttgagtgtc

S11wt-5

5334 gtgttggggtgagctgatctgaaatgagctactctggagtagctgagatggggtgagatggggtgagctgagctggg **Dir**

GTGTTGGGGTGAGCTGATCTGAAATGAGCTACGTATAGGTGACAGGATGGGGGAATTC**G**ACAAGGGC

9087 agagatccaagctgagcagctccagcttagctgtataggtgacaggatggggga-t--**a**acaa-ggc

S11wt-14

5142 ttgagccaaaatgaagtagactgtaatgaactggaatgagctgggccgctaagctaaactaggctggcttaaccga **Dir**

TTGAGCCAAAATGAAGTAGACTGTAATGAACTGGAATGATAATTGGCACGGGGTGGGGTGCATGCTGGGTACTCAT

8701 gtgtgggaacccagtcaaaaaccacagaagagcaggagctaattggcacggggtggggtgcatgctgggtactcat

S19wt-7

5308 ctggggtgagctcagctatgctacgc-gtgttggggtgagctgatctgaaatgagctactctggagtagctgagatg **Dir**

CTGGGGTGAGCTCAG-TATG-TACGCTGTGTTGGGGTGCTGAGCAG-TGAAGGTAACCTGGACCTAGTGGGggtgtg

8869 Ctacatagctggagctgatgggtgtataaggtaccaggctgagcagctgaaggtaacctgga**g**ctagtgggggtgtg

S9wt-103

5141 cttgagccaaaatgaagtagactgtaatgaactggaatgagctgggccgctaagctaaactaggctggcttaaccgag **Dir**

CTTGAGCCAAAATGAAGTAGACTGTAATGAACTGGAATGCTACTACATGAGAGCTGGAGCTAGTATGAAGGTGGAGGT

8967 ccaaggatcagggatagacatgtaagcagtcaagctcagctactacatgagagctggagctagtatgaaggtggaGGT

S9wt-101

5158 tagactgtaatgaactggaatgagctgggccgctaa**g**ctAAACTAGGCtggcttaaccgagatgagccaaactggaat **Dir**

TAGACTGTAATGAACTGGAATGAGCTGGGCCGCTAA**C**CTTGGGGATCCAGGTGCTGCAGCTACAGGTAAGCAGGGACAG

8389 GACagggaagctataggaaaaccaggacaggaggaagaatggggatccaggtgctgcagctacaggtaagcagggacag

S9wt-109

5375 GCTgagatggggtgagatggggtgagctgagctgggctGAGctggactgagctgagctagggtgagctgagctggG **Dir**

GCTGAGATGGGGTGAGATGGGGTGAGCTGAGCTGGGCTAGTTTAGTAGTTGTAGAGGAACAGGGGCAGATTAGAAt

8679 tccaggcagaacaggtccagggtgccaggacaggtacaagtttagtagttgtagaggaacaggggcagattagaat

S11wt-106

5270 TCtactgcctacactggactgttctgagctgagatgagctggggtgagctcagctatgctacgcgtgttg **Dir**

TCTACTGCCTACACTGGACTGTTCTTAACTGAGATGTAGAGAAACTGAGGCAAGTGGGAGTGCAGAGATC

9024 agctagtatgaaggtggaggtccagttgagtgtctttagagaaactgaggcaagtgggagtgcagagatc

S11wt-101

5151 aatgaagtagact**g**taatgaactggaatga**g**ctgggccgctaagctaaactaggctggcttaaccgaga **Dir**

AATGAAGTAGACT**T**TAATGAACTGGAATGA**T**CTGGGTTAGCTGGTATAGGTGACAGGATGGGGGA-A

9077 gtgggagtgcagagatccaagctgagcagctccagcttagct-gtataggtgacaggatgggggata

S11wt-104

5270 tctactgcctacactggactgttctgagctgagatgagctggggtgagctcagctatgctacgcgtgtt **Dir**

TCTACTGCCTACACTGGACTGTTCTGAGCT**T**AGATGGTATAGGTGACAGGA**G**GGGG**A**AT

9074 caagtgggagtgcagagatccaagctgagcagctccgtataggtgacagga**t**gggg**g**at

S11wt-113

5138 ggGcttgagccaaaatgaagtagactgtaatgaactggaatgagctgggccgctaagctaaactaggctg **Dir**

GCTTGAGCCAAAATGAAGTAGACTAGAATGCAGA-TCCAAACAGAAGAGCTACAGAGGAGCCAAGACA

8374 gctatagggcagccaggagaaatgGAagaatgcagattccaaacagaagagctacagaggagccaagaca

S19wt-101

5320 Cagctatgctacgc-gtgttggggtgagctgatctgaaatgagctactctggagtagctgagatggggtga **Dir**

CAGCTATGCTACGCTGTGTTGGGGTGAGCTGATCgagCTGGAGCTAGTATGAAGGTGGAGGTCCAGTTGAG

8983 gacatgtaagcagtcaagctcagctactacatgagagctggagctagtatgaaggtggaggtccagttgaG

S9wt-3

5173 tggaatgagctgggccgctaagctaaactaggctggcttaaccgagatgagccaaactggaatgaacttcattaatct **1nt**

TGGAATGAGCTGGGCCGCTAAGCTAAACTAGGCTGGCTTACCAGGCTGAGCAGCTGAAGGTAACCTGGACCTAGTGGGGG

8862 gcaaatactacatagctggagctgatgggtgtataaggtaccaggctgagcagctgaaggtaacctggagctagtggggg

S9wt-9

5151 aatgaagtagactgtaatgaactggaatgagctgggccgctaagctaaactaggctggcttaaccgagatgagccaaac **1nt**

AATGAAGTAGACTGTAATGAACTGGAATGAGCTGGGCCGCA**G**GCTGAGCAGCCCCAGCTTAGCTGGTATAGGTGACAGGAT

9055 gtctttagagaaactgaggcaagtgggagtgcagagatcca**a**gctgagcagc**t**ccagcttagct-gtataggtgacaggat

S19wt-2

5263 gctaaactctactgcctacactggactgttctgagctgagatgagctggggtgagctcagctatgctacgcgtgtTG **1nt**

TACACTGGACTGTTCTGAGCTGC**CA**GGACAGGTGGAAGTGTGG**T**GACCCAGGCAGAGCAGC

8421 ggaagaatggggatccaggtgctgcagctacaggtaagc**ag**ggacaggtggaagtgtgg**a**gacccaggcagagcagc

S19wt-12

5228 actggaatgaacttcattaatctaggttgaatagagctaaactctactgcctacactggactgttctgagctgAG **1nt**

ACTGGAATGAACTTCATTAATCTAGGTTGAATAG-GCTGTATAAGGTACCAGGCTGAGCAGCTGAAGGTAACCTG

8854 acagctgagcaaatactacatAgctggagctgatgggtgtataaggtaccaggctgagcagctgaaggtaACCTG

S19wt-104

5268 actctactgcctacactggactgttctgagctgagatgagctggggtgagctcagctatgctacgcgtgttg **1nt**

ACTCTACTGCCTACACTGGACTGTTCTGAGCTGagatATGAAGGTGGAGGTCCAGTTGAGTGTCTTTAGAGA

8994 agtcaagctcagctactacatgagagctggagctagtatgaaggtggaggtccagttgagtgtctttagaga

S19wt-105

5152 atgaagtagactgtaatgaactggaatgagctgggccgctaagctaaactaggctggcttaaccgagaTGAG **1nt**

ATGAAGTAGACTGTAATGAACTGGAATGAGCTGGGCCCAGTTGAGTGTCTTTAGAGAAACTGAGGCAAGTGG

9009 ctacatgagagctggagctagtatgaaggtggaggtccagttgagtgtctttagagaaactgaggcaagtgg

S19wt-107

5368 tggagtagctgagatggggtgagatggggtgagctgagctgggctgagctggactgagctgagctagggtga **1nt**

TGGAGTAGCTGAGATGGGGTGAGATGGGGTGAgctgATAGGTGACAGGATGGGGGA

9085 gcagagatccaagctgagcagctccagcttagctgtataggtgacaggatgggggA

S9wt-14

5159 agactgtaatgaactggaatgagctgggccgctaagctaaactaggctggcttaaccgagatgagccaaactggaatg **2nt**

AGACTGTAATGAACTGGAATGAGCTGGGCCGCTAAGCTAGAGAAACTGAGGCAAGTGGGAGTGCAGAGATCCA**G**GCtg

9023 gagctagtatgaaggtggaggtccagttgagtgtctttagagaaactgaggcaagtgggagtgcagagatcca**a**gCTG

S19wt-13

5219 atgagccaaactggaatgaacttcattaatctaggttgaatagagctaaactctactgcctacactggactgttc **2nt**

ATGAGCCAAACTGGAATGAACTTCATTAATCTAGGTTGAGCTGGAGCTAGTATGAAGGTGGAGGTCCAGttgagt

8979 atagacatgtaagcagtcaagctcagctactacatgagagctggagctagtatgaaggtggaggtccagttgagt

S9wt-110

5283 ctggactgttctgagctgagatga**g**ctggggtgagctcagctatgctacgcgtgttggggtgagctGatctgaaat **2nt**

CTGGACTGTTCTGAGCTGAGATGA**C**CTGGGGTGAGCTCAAGCTCAGCTACTACATGAGAGCTGGAGCTAGTATGAA

8960 gcagctaccaaggatcagggatagacatgtaagcagtcaagctcagctactacatgagagctggagctagtatgaa

S11wt-108

5231 gagccaaactggaatgaacttcattaatctaggttgaatagagctaaactctactgcctacactggactgt **2nt**

GAGCCAAACTGGAATGAACTTCATTAATCTAGGTTGAGAAACTGAGGCAAGTGGGAGTGCAGAGATCCAAG

9027 tagtatgaaggtggaggtccagttgagtgtctttagagaaactgaggcaagtgggagtgcagagatccaag

S11wt-112

5450 gtgagctgagct**g**agctggggtgagctgagctgagcTGagctgagctggggtgagctgagctgagctgggg **2nt**

GTGAGCTGAGCT**A**AGCTGGGGTGAGCTGAGCTGAGCAGCCAGGA**A**AGGTGGAAGTGTGG**G**G**CT**CCAGGCAG

4411 aatgtggtgacccaggcagagcagctccagggcagccagga**c**aggtggaagtgtgg**t**g**ac**ccaggcaG

S11wt-2

5223 gccaaactggaatgaacttcattaatctaggttgaatagagctaaactctactgcctacactggactgttctgagctg **3nt**

GCCAAACTGGAATGAACTTCATTAATCTAGGTTGAATAGATCCAAGCTGAGCAGCTCCAG**T**TTAGCTGGTATAGGTGA

9051 agtgtctttagagaaactgaggcaagtgggagtgcagagatccaagctgagcagctccag**c**ttagct-gtataggtgA

S11wt-1

5278 actctactgcctacactggactgttctgagctgagatgagctggggtgagctcagctatgctacgcgtgttggggtga **4nt**

ACTCTACTGCCTACACTGGACTGTTCTGAGCTGAGATGAGCTCCAGGTTAGCTGGTATAGGTGACAGGATGGGGGA-ATT

9068 ctgaggcaagtgggagtgcagagatccaagctgag--cagctccagcttagct-gtataggtgacaggatgggggata--

S11wt-114

5174 ggaatga**g**ctgggccgctaagctaaactag**g**ctggcttaaccgagatgagccaaactggaatgaacttca **4nt**

GGAATGA**A**CTGGGCCGCTAAGCTAAACTAG**A**CTGGCTGAGCAGCTGAAGGTAACCTGGAGCTAGTGGGGG

8872 catagctggagctgatgggtgtataaggtaccaggctgagcagctgaaggtaacctggagctagtGGGGG

S19wt-111

5311 gggtgagctcagctatgctacgc-gtgttggggtgagctgatctgaaatgagctactctggagtagctgagat **6nt**

GGGTGAGCTCAGCTATGCTACGCTGTGTTGGGGTGA**T**CTGaGCAGCTCCAGCTTAGCTGGTATAGGTGACAgg

9061 agagaaactgaggcaagtgggagtgcagagatccaagctgagcagctccagcttagct-gtataggtgacagg

***Aplf^–/–^***

S24APLFKO-11

MUSIGCD07 (Sμ)

5248 tctaggttgaatagagctaaactctactgcctacactggactgttctgagctgagatgagctggggtgagctcagct **Dir**

TCTAGGTTGAATAGAGCTAAACTCTACTGCCTACACTGCCAGTTGAGTGTCTTTAGAGAAACTGAGGCAAGTGGGAG

9007 tactacatgagagctggagctagtatgaaggtggaggtccagttgagtgtctttagagaaactgaggcaagtgggag

MUSIGHANB (Sγ1)

S25APLFKO-13

5140 gcttgagccaaaatgaagtagactgtaatgaactggaatgagctgggccgctaagctaaactaggctggcttaaccg **Dir**

GCTTGAGCCAAAATGAAGTAGACTGTAATGAACTGGAATGCAGCTGAGCAAATA-TACATAGCTGGAGCTGATGG**C**T

8815 agtcacagagaaactgatccaggtgagagtacggggtacacagctgagcaaatactacatagctggagctgatgg**g**t

S10APLFKO-101

5433 agggtgagctgagctgggtgagctgagct**g**agctggggtgagctgagctgagctgagctgagctggggtgagctgag **Dir**

agggtgagctgagctgggtgagctgagct**a**agctggggtagctccagcttagctggtataggtgacaggatggggga

9065 aaactgaggcaagtgggagtgcagagatccaagctgagcagctccagcttagct-gtataggtgacaggatggggga

S10APLFKO-102

5400 gctgagctgggctgagct**g**gactgagctgagctagggtgagctgagctgggtgagctgagctgagctggggtgagctg **Dir**

GCTGAGCTGGGCTGAGCT**A**GACTGAGCTGAGCTAGGGTGATTGAGTGTCTTTAGAGAAACTGAGGCAAGTGGGAGTGCAG

9009 ctacatgagagctggagctagtatgaaggtggaggtccagttgagtgtctttagagaaactgaggcaagtgggagtgcag

S10APLFKO-113

5184 gggccgctaagctaaactaggctggcttaaccgagatgagccaaactggaatgaacttcattaatctaggttgaatag **Dir**

GGGCCGCTAAGCTAAACTAGGCTGGCTTAACCGAGATGAGCATGAGAGCTGGAGCTAGTATGAAGGTGGAGGTCCAGTTGAG

8972 gatcagggatagacatgtaagcagtcaagctcagctactacatgagagctggagctagtatgaaggtggaggtccagttgag

S25APLFKO-103

5212 aaccgagatgagccaaactggaatgaacttcattaatcTaggttgaatagagctaaactctactgcctacactggact **Dir**

AACCGAGATGAGCCAAACTGGAATGAACTTCATTAATCTGCAAATACTACATAGCTGGAGCTGATGG**C**TGTATAAGGT

8823 agaaactgCTccaggtgagagtacggggtacacagctgagcaaatactacatagctggagctgatgg**g**tgtataaggt

S25APLFKO-107

5450 gtgagctgagctgagctggggtgagctgagctgagctgagctgagctggggtgagctgagctgagctggggtgagct **Dir**

GTGAGCTGAGCTGAGCTGGGGTGAGCTGAGCTGAGCTGTGGAGCTGATGG**C**TGTATAAGGTACCAGGCTGAGCAGCT

8840 agagtacggggtacacagctgagcaaatactacatagctggagctgatgg**g**tgtataaggtaccaggctgagcagct

S25APLFKO-110

5279 TAcactggactgttctgagctgagatgagctggggtgagctcagctatgctacgcgtgttggggtgagctgatctga **Dir**

TACACTGGACTGTTCTGAGCTGAGATGAGCTGGGGTGAAAGTTTAGTAGTTATAGAGGAACAGGGGCAGGTTAGAAT

3101 tcaaggcagaacaggtccaggggtgccaggacaggtgcaagttaagtacttatagaggaacaggggcaggttagaat

S224APLFKO_Sg1_6

5331 cGc-gtgttggggtgagctgatctgaaatgagctactctggagtagctgagatggggtgagatggggtgagctgagctgg **dir**

CGCTGTGTTGGGGTGAGCTGATCTGAAATGAGCTACTCTGTCAGGGATAGACATGTAAGCAGTCAAGCTCAGCTACTACA

8934 agtgggggtgtgggagaccaggctgagcagctaccaaggatcagggatagacatgtaagcagtcaagctcagctactaca

S224APLFKO_Sg1_13

5448 gggtgagctgagct**g**agctggggtgagctgagctgagctgagctgagctggggtgagctgagctgagctggggtgagc **dir**

gggTGAGCTGAGCT**A**AGCTGGGGTGAGCTGAGCTGAGCTTGACCCAGGCAGAGCAGCT**CC**AGGG**C**AGCCAGGACAggt

7734 cagagcagctatagggagccaggacaggtggaagtgtggtgacccaggcagagcagct**at**aggg**g**agccaggacaggt

S224APLFKO_Sg1_18

5156 agtagactgtaatgaactggaatgagctgggccgctaagctaaactaggctggcttaaccgagatgagccaaactgga **dir**

AGTAGACTGTAATGAACTGGAATGAGCTGGGCCGCTAAGAGTTGGGGATTCTAAGCAGTCACAGAGAAACTGATCCAG

8759 tgcatgctgggtactcatagggaagctgggataagtagtagttggggattctaagcagtcacagagaaactgatccag

S224APLFKO_Sg1_30

5151 aaatgaagtagactgtaatgaactggaatgagctgggccgctaagctaaactaggctggcttaaccgagatgagccaa **dir**

AAATGAAGTAGACTGTAATGAACTGGAATGAGCTGGGCCagTGGGGGTGTGGGAGACCAGGCTGAGCAGCTACCAAGG

8895 taaggtaccaggctgagcagctgaaggtaacctggagctagtgggggtgtgggagaccAggctgagcagctaccaagg

S224APLFKO_Sg1_32

5142 cttgagccaaaatgaagtagactgtaatgaactggaatgagctgggccgctaagctaaactaggctggcttaaccgag **dir**

CTTGAGCCAAAATGAAGTAGACTGTAATGAACTGGAATGAATA**G**GG**C**AGCCAG**G**ACAGGTGGGAGTGTGGGGATCCAG

8459 cagggacaggtggaagtgtggagacccaggcagagcagctata**a**gg**g**agccag**a**acaggtgggagtgtggggatccaG

S24APLFKO-2

5214 ccgagatgagccaaactggaatgaacttcattaatctaggttgaatagagctaaactctactgcctacactggactgt **1nt**

CCGAGATGAGCCAAACTGGAATGAACTTCATTAATCctgGACCTAGTGGGGGTGTGGGAGACC**T**GGCTGAGCAGCTACC

8890 Gtgtataaggtaccaggctgagcagctgaaggtaacctggagctagtgggggtgtgggagacc**a**ggctgagcagctacc

S24APLFKO-5

5276 gcctacactggactgttctgagctgagatgagctggggtgagctcagctatgctacgcgtgttggggtgagctgATC **1nt**

GCCTACACTGGACTGTTCTGAGCTGAGATGAGCTGGGGTGCTAGTATGAAGGTGGAGGTCCAGTTGAGTGTCTTTAG

8986 atgtaagcagtcaagctcagctactacatgagagctggagctagtatgaaggtggaggtccagttgagtgtctttag

S24APLFKO-12

5174 ggaatgagctgggccgctaagctaaactaggctggcttaaccgagatgagccaaactggaatgaacttcattaatc **1nt**

GGAATGAGCTGGGCCGCTAAGCTAAACTAGGCTGGGCAAGTGGGAGTGCAGAGATCCA**G**GCTGAGCAGCTCCAGCT

tggaggtccagttgagtgtctttagagaaactgaggcaagtgggagtgcagagatcca**a**gctgagcagctccagct

S25APLFKO-2

5158 tagactgtaatgaactggaatgagctgggccgctaagctaaactaggctggcttaaccgagatgagccaaactggaa **1nt**

TAGACTGTAATGAACTGGAATGAGCTGGGCCGCTAAGCTATCC**C**GGTGAGAGTACGGGGTACACAGCTGAGCAAATA

8792 agtagtagttggggattctaagcagtcacagagaaactgatcc**a**ggtgagagtacggggtacacagctgagcaaata

S25APLFKO-3

5272 tactgcctacactggactgttctgagctgagatgagctggggtgagctcagctatgctacgcgtgttggggtgag  **1nt**

TACTGCCTACACTGGACTGTTCTGAGCTGAGATGAG--GGTATAGGTGACAGGATGGGGGA-ATTCGACAAGGGC

9080 GgagtgcagagatccaagctgagcagctccagcttagctgtataggtgacaggatgggggaTA----ACAA-GGC

S10APLFKO-114

5179 gagctgggccgctaagctaaactaggctggcttaaccgagatgagccaaactggaatgaacttcattaatctaggttg **1nt**

GAGCTGGGCCGCTAAGCTAAACTAGGCTGGCTTAACCGAGACCAGGACAGG**T**GGAAGAATGGGGATCCAGGTGCTGCAGcta

8369 agcagtaccttaggagcaaggacagggaagctataggaaaaccaggacagg**a**ggaagaatggggatccaggtgctgcagcta

S24APLFKO-101

5501 tgagctg**a**gctgagctggggtgagctgagctgagctggggtgagctgagctgagct **1nt**

TGAGCTG**G**GCTGAGCTGGGGTGAGCTGAGCTGAGCTGGGGTGCAGAACAGGTCCA-GGGTGCCAGGACAGGT**A**CAAGTT**T**AG

3065 gctataggggggccaagacaggtggaagtgtggggatcaaggcagaacaggtccaggggtgccaggacaggt**g**caagtt**a**ag

S24APLFKO-106

5273 actgcctacactggactgttctga**g**ctgagatgagctggggtgagctcagctatgctacgcgtgttggggtgagctga **1nt**

ACTGCCTACACTGGACTGTTCTGA**A**CTGAGATGAGCTGGGGCTAATTGGCACGGGCTGGGGTGCATGCTGG**CA**ACTCATAG

8698 Agagtgtgggaacccagtcaaaaaccacagaagagcaggagctaattggcacggggtggggtgcatgctgg**gt**actcatag

S24APLFKO-110

5334 gtgttggggtgagctgatctgaaatgagctactctggagtagctgagatggggtgagatggggtgagctgagctgggc **1nt**

GTGTTGGGGTGAGCTGATCTGAAATGAGATACTCTGGAATTGGCACGGGCTGGGGTGCATGCTGGCTACTCATAGGGAAG

8704 tgggaacccagtcaaaaaccacagaagagcaggagctaattggcacggggtggggtgcatgctgggtactcatagggaag

S25APLFKO-104

5254 ttgaatagagctaaactctactgcctacactggactgttctgagctgagatgagctggggtgagctcagctatgctac **1nt**

TTGAATAGAGCTAAACTCTACTGCCTACACTGGACTGTTATGAAGGTGGAGGTCCAGTTGAGTGTCTTTAGAGAAACT

8992 gcagtcaagctcagctactacatgagagctggagctagtatgaaggtggaggtccagttgagtgtctttagagaaact

S224APLFKO_Sg1_9

5246 aatctaggttgaatagagctaaactctactgcctacactggactgttctgagctgagatgagctggggtgagctcagcta **1nt**

AATCTAGGTTGAATAGAGCTAAACTCTACTGCCTACACTGt-gTAGTTGGGGATTCTAAGCAGTCACAGAGAAACTGATC

8754 tggggtgcatgctgggtactcatagggaagctgggataagtagtagttggggattctaagcagtcacagagaaactgatc

S224APLFKO_Sg1_17

5364 actctggagtagctgagatggggtgagatggggtgagctgagctgggctgagctggactgagctgagctagggtgagc **1nt**

ACTCTGGAGTAGCTGAGATGGGGTGAGATGGGGTGAGCTGTACCAGGCTGAGCAGCTGAAGGTAACCTGGAGCTAGTG

8860 gagcaaatactacatagctggagctgatgggtgtataaggtaccaggctgagcagctgaaggtaacctggagctagtg

S224APLFKO_Sg1_29

5411 gctgagctggactgagctgagctagggtgag**c**t**g**agctgggtgagctgagctgagctggggtgagctgagc **1nt**

GCTGAGCTGGACTGAGCTGAGCTAGGGTGAG**T**T**T**AGCTGGTATAGGTGACAGGA**G**GGGG**A**ATT**CG**ACAAGG

9080 ggagtgcagagatccaagctgagcagctccagcttagctgtataggtgacagga**t**gggg**g**at—-**a**acaagg

S224APLFKO_Sg1_38

5371 Gagtagctgagatggggtgagatggggtgagctgagctgggctgagctggactgagctgagctagggtgagc **1nt**

GAGTAGCTGAGATGGGGTGAGATGGGGTGAGCTGAG-TGGTATAGGTGACAGGATGGGGGA-ATTCGACAAGG

9080 ggagtgcagagatccaagctgagcagctccagCttagctgtataggtgacaggatgggggata----acaagG

S10APLFKO-10

5140 gcttgagccaaaatgaagtagactgtaatgaactggaatgagctgggccgctaagctaaactaggctggcttaa **2nt**

gcttgagccaaaatgaagtagactgtaatgaactggaatgtataggtgacaggatggggga-attcgacaagg**g**

9081 GGAgtgcagagatccaagctgagcagctccagcttagctgtataggtgacaggatgggggata----acaagg**c**

S10APLFKO-103

5243 attaatctaggttgaatagagctaaactctactgccta**c**actggactgttctgagctgagatgagctggggtgagctc **2nt**

ATTAATCTAGGTTGAATAGAGCTAAACTCTACTGCCTA**T**ACAG**G**TGG**G**AGTGTGGTGACCC**A**GGCAGAGCA**T**CTATAGG

4265 ttgtggtgacccaggcagagcagctccaggggagccaggacag**t**tgg**a**agtgtggtgaccc**t**ggcagagca**g**ctatagg

S10APLFKO-108

5501 Tgagctgagctgagctggggtgagctgagctgagctggggtgagctgagctgagct **2nt**

TGAGCTGAGCTGAGCTGGGGTGAGCTGAGCTGAGCTGGGGTCAAGCTCAGCTACTACATGAGAGCTGGAGCTAGTATGAA

8956 ctgagcagctaccaaggatcagggatagacatgtaagcagtcaagctcagctactacatgagagctggagctagtatgaa

S24APLFKO-104

5187 cgctaagctaaactaggctggcttaaccgagatgagccaaactggaatgaacttcattaatctaggttgaatagagct **2nt**

cgCTAAGCTAAACTAGGCTGGCTTAACCGAGATGAGCC-AACTACCAAGGATCAGGGATAGACATGTAAGCAGTCA**G**GCTc

8923 aacctggagctagtgggggtgtgggagaccaggctgagcagctaccaaggatcagggatagacatgtaagcagtca**a**gctc

S25APLFKO-105

5263 gctaaactctactgcctacactggactgttctgagctgagatgagctggggtgagctcagctatgctacgcgtgttG **2nt**

GCTAAACTCTACTGCCTACACTGGACTGTTCTGAGCTGGGAGTGCAGAGATCCA**G**GCTGAGCAGCTCCAGCTTAGCT

9042 ggtccagttgagtgtctttagagAaactgaggcaagtgggagtgcagagatcca**a**gctgagcagctccagcttagct

S224APLFKO_Sg1_1

5263 gctaaactctactgcctacactggactgttctgagctgagatgagctggggtgagctcagctatgctacgcgt **2nt**

GCTAAACTCTACTGCCTACACTGGACTGTTCTGAGCTGAGCTTAG**G**TGGTATAGGTGACAGGATGGGGGA-a

9072 ggcaagtgggagtgcagagatccaagctgagcagctccagcttag**c**t-gtataggtgacaggatgggggata

S224APLFKO_Sg1_19

5347 ctgatctgaaatgag**c**tactctggagtagctgagatggggtgagatggggtgagctgagctgggctgagctggactg **2nt**

CTGATCTGAAATGAG**A**TACTCTGGAGTAGCTGAGATGGgA**C**CTAGTGGGGGTGTGGGAGACC**T**GGCTGAGCAGCTAc

8891 tgtataaggtaccaggctgagcagctgaaggtaacctgga**g**ctagtgggggtgtgggagacc**a**ggctgagcagctaC

S10APLFKO-1

5223 gccaaactggaatgaacttcattaatctaggttgaatagagctaaactctactgcctacactggactgttctgagCT **3nt**

GCCAAACTGGAATGAACTTCATTAATCTAGGTTGAATAGATCCAAGCTGAGCAGCTCCAG**T**TTAGCTGGTATAGGTG

9052 agtgtctttagagaaactgaggcaagtgggagtgcAgagatccaagctgagcagctccag**c**ttagct-gtataggTG

S224APLFKO_Sg1_14

5223 Gccaaactggaatgaacttcattaatctaggttgaatagagctaaactctactgcctacactggactgttctgagctg **3nt**

GCCAAACTGGAATGAACTTCATTAATCTAGGTTGAATAGAGTGCAGAGATCCAAGCTGAGCAGCTCCAGCTTAGCtgg

9043 gtccagttgagtgtctttagagaaaCtgaggcaagtgggagtgcagagatccaagctgagcagctccagcttagct-G

S25APLFKO-10

5143 tgagccaaaatgaagtagactgtaatgaactggaatgagctgggccgctaagctaaactaggctggcttaaccgagat **4nt**

TGAGCCAAAATGAAGTAGACTGTAATGAACTGGAATGAGCTACCAAGGATCAGGGATAGACATGTAAGCAGTCAAGCT

8925 cctggagctagtgggggtgtgggagaccaggctgagcagctaccaaggatcagggatagacatgtaagcagtcaagct

S25APLFKO-11

5402 tgagctgggctgagct**g**gactgagctgagctagggtgagctgagctgggtgagctgagctgagctggggtgagctgag **4nt**

TGAGCTGGGCTGAGCT**A**GACTGAGCTGAGCTAGGGTGAGCTATAGGGAGCCAGGACAGGTGGAAGTGTG**T**TGA**T**CCAG

7917 gccaggacaggtggaaatgtggtgacccaggcagagtagctatagggagccaggacaggtggaagtgtg**g**tga**c**ccag

S224APLF_Sg1_12

5478 agctgagc-tgagctgagct**g**gggtgagctg**ag**ctgagctggggtgagctgagctgagctggggtgagctgagctgag **4nt**

AGCTGAGCTTGA-CTGAGCT**A**GGGTGAGCTG**GA**CTGAGCTGCAGCTACAGGTAAGCAGGGACAGGTGGAAGTGTGGAG

8404 ggaaaaccaggacaggaggaagaatggggatccaggtgctgcagctacaggtaagcagggacaggtggaagtgtggaG

S224APLFKO_Sg1_4

5216 gagatgagccaaactggaatgaacttcattaatctaggttgaatagagctaaactctactgcctacactggactgttctg **5nt**

GAGATGAGCCAAACTGGAATGAACTTCATTAATCTAGGTTGAGTGTCTTTAGAGAAACTGAGGCAAGTGGGAGTGCAGAG

9011 acatgagagctggagctagtatgaaggtggaggtccagttgagtgtctttagagaaactgaggcaagtgggagtgcagag

S224APLFKO_Sg1_8

5365 Ctctggagtagctgagatggggtgagatggggtgagctgagctgggctgagctggactgagctgagctagggttggggtg **5nt**

CTCTGGAGTAGCTGAGATGGGGTGAGATGGGGTGAGCTGAGCTAGTATGAAGGTGGAGGTCCAGTTGAGTGTCTTTAGAG

8985 catgtaagcagtcaagctcagctactacatgagagctggagctagtatgaaggtggaggtccagttgagtgtctttagaG

S10APLFKO-104

5392 tggggtgagctgagctgggctgagct**g**gactgagctgagctagggtgagctgagctgggtgagctgagctgagctggG **6nt**

TGGGGTGAGCTGAGCTGGGCTGAGCT**A**GACTGAGCTGAGCTAGTGGGGGTGTGGGAGACC**T**GGCTGAGCAGCTACCAag

8893 tataaggtaccaggctgagcagctgaaggtaacctggagctagtgggggtgtgggagacc**a**ggctgagcagctaccaaG

S25APLFKO-111

5226 aaactggaatgaacttcattaatctaggttgaatagagctaaactctactgcctacactggactgttctgagctgaga **7nt**

AAACTGGAATGAACTTCATTAATCTAGGTTGAATAGAGCTAATTGGCACGGG**C**TGGGGTGCATGCTGG**C**TACTCATAGG

8701 GTGTgggaacccagtcaaaaaccacagaagagcaggagctaattggcacggg**g**tggggtgcatgctgg**g**tactcatagg

***Atm^–/–^***

S28ATMKO_Sg1__101

MUSIGCD07 (Sμ)

5272 tactgcctacactggactgttctgagctgagatgagctggggtgagctcagctatgctacgtgttggggtgagctgatc **dir**

TACTGCCTACACTGGACTGTTCTGAGCTGAGATGAGCTGGGAGGACAGGTGGAAATGTGGTGACCCAGGCAGAGTAGCT

7196 tggaagtgtggtgacccaggcagagcagctataggagagccaggacaggtggaaatgtggtgacccaggcagagtagct

MUSIGHANB (Sγ1)

S28ATMKO_Sg1__120

5158 tagactgtaatgaactggaatgagctgggccgctaagctaaactaggctggcttaaccgagatgagccaaactggaatg **dir**

TAGACTGTAATGAACTGGAATGAGCTGGGCCGCTAAGCTAATATAAGGTACCAGGCTGAGCAGCTGAAGGTAACCTGGA

8852 acacagctgagcaaatactacatagctggagctgatgggtgtataaggtaccaggctgagcagctgaaggtaacctgga

S28ATMKO_Sg1_206

5272 tactgcctacactggactgttctgagctgagatgagctggggtgagctcagctatgctacgcgtgttggggtgagctga **dir**

TACTGCCTACACTGGACTGTTCTGAGCTGAGATGAGCTGAATTGGCACGGG**C**TGGGGTGCATGCTGG**C**TACTCATAGGG

8701 tgtgggaacccagtcaaaaaccacagaagagcaggagctaattggcacggg**g**tggggtgcatgctgg**g**tactcataggg

S28ATMKO_Sg1_207

5356 aatgagctactctggagtagctgagatggggtgagatggggtgagctgagctgggctgagctggactgagctgagctag **dir**

AATGAGCTACTCTGGAGTAGCTGAGATGGGGTGAGATGGCAGGGAAGCTATAGGAAAACCAGGACAGGAGGAAGAATGG

8352 tgtgtgaatccaggcagagcagtaccttaggagcaaggacagggaagctataggaaaaccaggacaggaggaagaatgg

S4ATMKO_Sg1_101

5280 acactggactgttctgagctgagatgagctggggtgagctcagctatgctacgcgtgttggggtgagctgatctgaaatg **dir**

acactggactgttctgagctgagatgagctggggtgagcttagtatgaaggtggaggtccagttgagtgtctttagagaa

8988 tgtaagcagtcaagctcagctactacatgagagctggagctagtatgaaggtggaggtccagttgagtgtctttagagaa

S4ATMKO_Sg1_102

5267 aactctactgcctacactggactgttctgagctgagatgagctggggtgagctcagctatgctacgcgtgttggggtgag **Dir**

aactctactgcctacactggactgttctgagctgagatgaatgagagctggagctagtatgaaggtggaggtccagttga

8973 atcagggatagacatgtaagcagtcaagctcagctactacatgagagctggagctagtatgaaggtggaggtccagttga

S14ATMKO_Sg1_112

5274 ctgcctacactggactgttctgagctgagatgagc**t**ggggtgagctcagctatgctacgcgtgttggggtgagctgatct **Dir**

ctgcctacactggactgtt-tgag-tga-atga--**g**ggggagctcagctactacatgagagctggagctagtatgaaggt

8959 agcagctaccaaggatcagggatagacatgtaagcagtcaagctcagctactacatgagagctggagctagtatgaaggt

S22ATMKO_Sg1_204

5345 agctgatctgaaatgag**c**tactctggagtagctgagatggggtgagatggggtgagctgagctgggctgagctggactga **Dir**

agctgatctgaaatgag**a**tactctggagtagctgagatggaggggcaggttagaatgaaggatg**g**gcatcccgggtgagc

3122 ggtgccaggacaggtgcaagttaagtacttatagaggaacaggggcaggttagaatgaaggatg**t**gcatcccgggtgagc

S22ATMKO_Sg1_216

5256 gaatagagctaaactctactgcctacactggactgttctgagctgagatgagctggggtgagctcagctatgctacgc **dir**

gaatagagctaaactctactgcctacactggactgttctccacagaagagcaggagctaattggcacggg**c**tggg**a**tg

8683 aagctgaggcaggtaagagtgtgggaacccagtcaaaaaccacagaagagcaggagctaattggcacggg**g**tggg**g**tg

S22ATMKO_Sg1_220

5409 ggctgagct**g**gactgagctgagctagggtgagctg**a**gctgggtgagctgagctgagctggggtgagctgagctgagctga **dir**

ggctgagct**a**gactgagctgagctagggtgagctg**g**gctgagctactacatgagagctggagctagtatgaaggtggagg

8964 ctaccaaggatcagggatagacatgtaagcagtcaagctcagctactacatgagagctggagctagtatgaaggtggagg

S22ATMKO_Sg1_34

5346 gctgatctgaaatgag**c**tactctggagtagctgagatggggtgagatggggtgagctgagctgggctgagctggactga **dir**

GCTGATCTGAAATGAG**A**TACTCTGGAGTAGCTGAGATGGGAGTCAAAAACCACAGAAGAGCAGGAGCTAATTGGCACGG

8673 agatacagggaagctgaggcaggtaagagtgtgggaacccagtcaaaaaccacagaagagcaggagctaattggcacgg

S28ATMKO_Sg1_205

5454 gctgagctgagctgg**g**gtgagctgagctg**a**gctgagctg**a**gctggggtgagctgagctgagctggggtgagctgagctga **dir**

gctgagctgagctgg**a**gtgagctgagctg**g**gctgagctg**g**ggtgtggagacccaggcagagcagctataagggagccaga

8433 atccaggtgctgcagctacaggtaagcagggacaggtggaagtgtggagacccaggcagagcagctataagggagccaga

S28ATMKO_Sg1_219

5503 agctgagctgagctgg**g**gtgagctgagctg**a**gctg**g**g**g**tg**a**gctgagctgagct **DIr**

agctgagctgagctgg**a**gtgagctgagctg**g**gctg**a**g**c**tg**g**ggtgtggagacccaggcagagcagctataagggagccagaa

8432 gatccaggtgctgcagctacaggtaagcagggacaggtggaagtgtggagacccaggcagagcagctataagggagccagaa

S28ATMKO_Sg1__102

5458 agct**g**agctggggtgagctgagctgagct**ga**gctgagctggggtgagctgagctgagctggggtgagctgagct **1nt**

AGCT**A**AGCTGGGGTGAGCTGAGCTGAGCT**TG**GCTGAGCTGGTATAGGTGACAGGATGGGGGA-ATTCGACAAGG

9079 gggagtgcagagatccaagctgagcagctccagcttagctgtataggtgacaggatgggggata----acaagg

S28ATMKO_Sg1_205

5250 taggttgaatagagctaaactctactgcctacactggactgttctgagctgagatgagctggggtgagctcagctatgc **1nt**

TAGGTTGAATAGAGCTAAACTCTACTGCCTACACTGGACAAGGTAGGTGGAATGTGAATATCCAGGCAGAACAGGTCCA

8519 gggagtgtggggatccaggtaaggctggactggggagccaaggtaggtggaatgtgaatatccaggcagaacaggtcca

S28ATMKO_Sg1_216

5515 tggggtgagctg**a**gctgagctg**g**g**g**tgagctgagctgagct **1nt**

tggggtgagctg**g**gctgagctg**a**g**c**tgagctgagctgagctgagctgagctgagctg**a**g**c**tgagctgagctgagctga

5411 ctgagctggactgagctgagctagggtgagctgagctgggtgagctgagctgagctg**g**g**g**tgagctgagctgagctga

S14ATMKO-Sg1-2

5253 gttgaatagagctaaactctactgcctacactggactgttctgagctgagatgagctggggtgagctcagctatgctacg **1nt**

GTTGAATAGAGCTAAACTCTACTGCCTACACTGGACTgttcAG**A**GAAGCTGAGGCAGGTAAGAGTGTGGGAACCCAGTCA

8638 caggggcagattagaatgaatgcacctgggtgagcagatacag**g**gaagctgaggcaggtaagagtgtgggaacccagtca

S14ATMKO_Sg1_109

5140 gcttgagccaaaatgaagtagactgtaatgaactggaatgagctgggccgctaagctaaactaggctggcttaaccgaga **1nt**

gcttgagccaaaatgaagtagactgtaatgaactggaatgctactacatgagagctggagctagtatgaaggtggaggtc

8966 accaaggatcagggatagacatgtaagcagtcaagctcagctactacatgagagctggagctagtatgaaggtggaggtc

S22ATMKO_Sg1_217

5282 actggactgttctgagctgagatgagctggggtgagctcagctatgctacgcgtgttggggtgagctgatctgaaatg **1nt**

actggactgttctgagctgagatgagctggggtgagctctgatgg**c**tgtataaggtaccaggctgagcagctgaaggt

8845 acggggtacacagctgagcaaatactacatagctggagctgatgg**g**tgtataaggtaccaggctgagcagctgaaggt

S22ATMKO_Sg1_27

5150 aaatgaagtagactgtaatgaactggaatgagctgggccgctaagctaaactaggctggcttaaccgagatgagccaaac **1nt**

AAATGAAGTAGACTGTAATGAACTGGAATGAGCTGGGCCGGTGAGAGTACGGGGTACACAGCTGAGCAAATACTACATAG

8797 tagttggggattctaagcagtcacagagaaactgatccaGgtgagagtacggggtacacagctgagcaaatactacatag

S22ATMKO_Sg1_35

5373 tagctgagatggggtgagatggggtgagctgagctgggctgagctggactgagctgagctagggtgagctgagctgggt **1nt**

TAGCTGAGATGGGGTGAGATGGGGTGAGCTGAGCTGGGCTACTCATAGGGAAGCTGGGATAAGTAGTAGTTGGGGATTC

8731 agcaggagctaattggcacggggtggggtgcatgctgggtactcatagggaagctgggataagtagtagttggggattc

S22ATMKO_Sg1_46

5421 ctgagctgagctagggtgagctgagctgggtgagctgagctgagctggggtgagctgagctgagctgagctgagctggg **1nt**

CTGAGCTGAGCTAGGGTGAGCTGAGCTGGGTGAGCTGAGCTacTCATAGGGAAGCTGGGATAAGTAGTAGTTGGGGATT

8770 gagcaggagctaattggcacggggtggggtgcatgctgggtactcatagggaagctgggataagtagtagttggggatt

S22ATMKO_Sg1_53

5251 aggttgaatagagctaaactctactgcctacactggactgttctgagctgagatgagctggggtgagctcagctatgct **1nt**

AGGTTGAATAGAGCTAAACTCTACTGCCTACACTGGACTgtAGCTGGAGCTGATGG**C**TGTATAAGGTACCAGGCTGAGC

8834 caggtgagagtacggggtacacagctgagcaaatactacatagctggagctgatgg**g**tgtataaggtaccaggctgagc

S28ATMKO_Sg1_206

5303 atgagctGgggtgagctcagctatgctacgc-gtgttggggtgagctgatctgaaatgagctactctggagtagctgaga **1nt**

atgagctggggtgagctcagctatgctacgctgtgttggggtacatagctggagctgatgggtgtataaggtaccaggct

8829 tgatccaggtgagagtacggggtacacagctgagcaaatactacatagctggagctgatgggtgtataaggtaccaggct

S28ATMKO_Sg1_209

5296 agctGagatgagctggggtgagctcagctatgctacgc-gtgttggggtgagctgatctgaaatgagctactctggagta **1nt**

agctgagatgagctggggtgagctcagctatgctacgctgtggtggaggtccagttgagtgtctttagagaaactgaggc

8995 gtcaagctcagctactacatgagagctggagctagtatgaaggtggaggtccagttgagtgtctttagagaaactgaggC

S28ATMKO_Sg1_210

5148 Caaaatgaagtagactgtaatgaactggaatgagctgggccgctaagctaaactaggctggcttaaccgagatgagcca **2nt**

CAAAATGAAGTAGACTGTAATGAACTGGAATGAGCTGGGCtGAGCAGCTCCAGCTTAGCTGGTATAGGTGACAGGATGG

9059 ttagagaaactgaggcaagtgggagtgcagagatccaagctgagcagctccagcttagct-gtataggtgacaggatgg

S28ATMKO_Sg1_236

5213 Accgagatgagccaaactggaatgaacttcattaatctaggttgaatagagctaaactctactgcctacactggactgt **2nt**

ACCGAGATGAGCCAAACTGGAATGAACTTCATTAATCTAGGACAGGTGGAAGTGTGGAGACCCAGGCAGAGCAGCTATA

8423 aagaatggggatccaggtgctgcagctacaggtaagcagggacaggtggaagtgtggagacccaggcagagcagctata

S4ATMKO-Sg1-2

5162 ctgtaatgaactggaatgagctgggccgctaagctaaactaggctggcttaaccgagatgagccaaactggaatgaactt **2nt**

CTGTAATGAACTGGAATGAGCTGGGCCGCTAAGCTAAACCAGGCTGAGCAGCTGAAGGTAACCTGGA**C**CTAGT-GGGGTG

8864 aaatactacatagctggagctgatgggtgtataaggtaccaggctgagcagctgaaggtaacctgga**g**ctagtgggggtg

S4ATMKO-Sg1-19

5212 Aaccgagatgagccaaactggaatgaacttcattaatctaggttgaatagagctaaactctactgcctacactggactgt **2nt**

AACCGAGATGAGCCAAACTGGAATGAACTTCATTAATCTAGGAGCTGATGG**C**TGTATAAGGTACCAGGCTGAGCAGCTGA

8839 gagagtacggggtacacagctgagcaaatactacatagctggagctgatgg**g**tgtataaggtaccaggctgagcagctga

S4ATMKO_Sg1_123

5223 gccaaactggaatgaacttcattaatctaggttgaatagagctaaactctactgcctacactggactgttctgagctgag **2nt**

gccaaactggaatgaacttcattaatctaggttgaatagaggtcca-gggtgccaggacaggt**a**caagtt**t**agta**g**ttat

3074 Gggccaagacaggtggaagtgtggggatcaaggcagaacaggtccaggggtgccaggacaggt**g**caagtt**a**agta**c**ttat

S22ATMKO_Sg1_201

5339 ggggtgagctgatctgaaatgag**c**tactctggagtagctgagatggggtgagatggggtgagctgagctgggctgagctg **2nt**

ggggtgagctgatctgaaatgag**a**tactctggagtagctgaacaggtcca-gggtgccaggacaggt**a**caagtttagtag

3070 aggggggccaagacaggtggaagtgtggggatcaaggcagaacaggtccaggggtgccaggacaggt**g**caagttaagtac

S22ATMKO_Sg1_205

5221 gagccaaactggaatgaacttcattaatctaggttgaatagagctaaactctactgcctacactggactgttctgagctg **2nt**

gagccaaac-ggaatgaacttca-taat-taggttgaatacatagctggagctgatgg**c**tgtataaggtaccaggctgag

8832 tccaggtgagagtacggggtacacagctgagcaaatactacatagctggagctgatgg**g**tgtataaggtaccaggctgag

S22ATMKO_Sg1_206

5485 ctgagctgagctggggt**g**agctg**a**g**c**tgagctggggtgagctgagctgagctggggtgagctgagctgagct **2nt**

ctgagctgagctggggt**a**agctg**g**g**a**tgagctggggtgacaggtggaagtgtggagacccaggcagagcagctata**g**g

8426 aatggggatccaggtgctgcagctacaggtaagcagggacaggtggaagtgtggagacccaggcagagcagctata**a**g

S22ATMKO_Sg1_214

5143 tgagccaaaatgaagtagactgtaatgaactggaatgagctgggccgctaagctaaactaggctggcttaaccgagat **2nt**

tgagccaaaatgaagtagactgtaatgaactggaatgagcaggttagaatgaaggatg**g**gcatcccgggtgagcaaat

3128 aggacaggtgcaagttaagtacttatagaggaacaggggcaggttagaatgaaggatg**t**gcatcccgggtgagcaaat

S22ATMKO_Sg1_29

5214 ccgagatgagccaaactggaatgaacttcattaatctaggttgaatagagctaaactctactgcctacactggactgttc **2nt**

CCGAGATGAGCCAAACTGGAATGAACTTCATTAATCTAGgAGCTGATGG**C**TGTATAAGGTACCAGGCTGAGCAGCTGAAG

8841 gagtacggggtacacagctgagcaaatactacatagctggagctgatgg**g**tgtataaggtaccaggctgagcagctgaag

S22ATMKO_Sg1_38

5178 tgagctgggccgctaagctaaactaggctggcttaaccgagatgagccaaactggaatgaacttcattaatctaggttg **2nt**

TGAGCTGGGCCGCTAAGCTAAACTAGGCTGGCTTAACCgagCT**A**AGCTGGTATAGGTGACAGGATGGGGGA-attcgac

9071 aggcaagtgggagtgcagagatccaagctgagcagctccAgct**t**agct-gtataggtgacaggatgggggata----ac

S28ATMKO_Sg1_202

5142 ttgagccaaaatgaagtagactgtaatgaactggaatgagctgggccgctaagctaaactaggctggcttaaccgagatg **2nt**

ttgagccaaaatgaagtagactgtaatgaactggaatgagctcatagggaagctgggataagtagtagttggggattcta

8732 gcaggagctaattggcacggggtggggtgcatgctgggtactcatagggaagctgggataagtagtagttggggattctA

S28ATMKO_Sg1__110

5301 Agatgagctggggtgagctcagctatgctacgc-gtgttggggtgagctgatctgaaatgagctactctggagtagctga **3nt**

AGATGAGCTGGGGTGAGCTCAGCTATGCTACGCTGTGTTgggATAGACATGTAAGCAGTCAAGCTCAGCTACTACATGAG

8938 ggggtgtgggagaccaggctgagcagctaccaaggatcagggatagacatgtaagcagtcaagctcagctactacatgag

S28ATMKO_Sg1 114

5243 attaatctaggttgaatagagctaaac**t**ctactgcctacactggactgttctgagctgagatgagctggggtgagctca **3nt**

ATTAATCTAGGTTGAATAGAGCTAAAC**C**CTACTGCCTACACTACATGAGAGCTGGAGCTAGTATGAAGGTGGAGGTCCA

8969 aaggatcagggatagacatgtaagcagtcaagctcagctactacatgagagctggagctagtatgaaggtggaggtcca

S28ATMKO_Sg1_204

5243 attaatctaggttgaatagagctaaactctactgcctacactggactgttctgagctgagatgagctggggtgagctca **3nt**

ATTAATCTAGGTTGAATAGAGCTAAACTCTACTGCCTACAgGTGGGAGTGTGGGGATCCAGGTAAGGC**A**GGACTGGGGA

8476 gtggagacccaggcagagcagctataagggagccagaacaggtgggagtgtggggatccaggtaaggc**t**ggactgggga

S4ATMKO-Sg1-1

5293 CTGagctgagatgagctggggtgagctcagctat-gctacgcgtgttggggtgagctgatctgaaatgagctactctgga **3nt**

CTGAGCTGAGATGAGCTGGGGTGAGCTCAGCTATGGCTACAG**A**GAAGCTGAGGCAGGTAAGAGTGTGGGAACCCAGTCAA

8639 aggggcagattagaatgaatgcacctgggtgagcagatacag**g**gaagctgaggcaggtaagagtgtgggaacccagtcaa

S14ATMKO-Sg1-1

5339 ggtgagctgatctgaaatgag**c**tactctggagtagctgagatggggtgagatggggtgagctgagctgggctgagctgga **3nt**

GGTGAGCTGATCTGAAATGAG**A**TACTCTGGAGTAGCTGAGATCAGGGATAGACATGTAAGCAGTCAAGCTCAGCTACTAC

8933 tagtgggggtgtgggagaccaggctgagcagctaccaaggatcagggatagacatgtaagcagtcaagctcagctactac

S14ATMKO-Sg1-6

5266 Aaactctactgcctacactggactgttctgagctgagatgagctggggtgagctcagctatgctacgcgtgttggggtg **3nt**

AAACTCTACTGCCTACACTGGACTGTTCTGAGCTGAGATGATGG**C**TGTATAAGGTACCAGGCTGAGCAGCTGAAGGTAA

8846 cggggtacacagctgagcaaatactacatagctggagctgatgg**g**tgtataaggtaccaggctgagcagctgaaggtaa

S14ATMKO-Sg1-18

5195 ctaaactaggctggcttaaccgagatgagccaaactggaatgaacttcattaatctaggttgaatagagctaaactct **3nt**

ct-AACTAGGCTGG-ttaa-CGAGATGAGCCAAACTGGAAga-caggag-TAATTGGCAC--GGTGGGGTGCATGCTg

8690 ggcaggtaagagtgtgggaacccagtcaaaaaccacagaagagcaggagctaattggcacggggtggggtgcatgctg

S14ATMKO_Sg1_101

5503 agctgagctgagct**g**gggtgagctgagctg**a**gctggggtgagctgagctgagct **3nt**

agctgagctgagct**a**gggtgagctgagctg**t**gctggggtgatcc**c**ggtgagagtacggggtacacagctgagcaaatact

8491 gagcagctataagggagccagaacaggtggagagaaactgatcc**a**ggtgagagtacggggtacacagctgagcaaatact

S22AMTKO_Sg1_208

5221 gagccaaactggaatgaacttcattaatctaggttgaatagagctaaactctactgcctacactggactgttctgagct **3nt**

gagccaaactggaatgaacttcattaatctaggttgaataa-tagtagttggggattctaagcagtcacagagaa**G**ctg

8752 ggtggggtgcatgctgggtactcatagggaagctgggataagtagtagttggggattctaagcagtcacagagaa**A**ctg

S22ATMKO_Sg1_37

5282 actggactgttctgagctgagatgagctggggtgagctcagctatgctacgcgtgttggggtgagctgatctgaaatga **3nt**

ACTGGACTGTTCTGAGCTGAGATGAGCTGGGGTGAGCTCAGAGGAGCCAAGACAACTAGAAGTGTGTGAATCCAGGCAG

8290 gagaaatggaagaatgcagattccaaacagaagagctacagaggagccaagacaactagaagtgtgtgaatccaggcag

S28ATMKO_Sg1_207

5477 gagctGagc-tgagctgagct**g**gggtgagctg**ag**ctgagctggggtgagctgagctgagctggggtgagctgagctgagc **3nt**

gagctgagcttga-ctgagct**a**gggtgagctg**ga**ctgagctggaggtccagttgagtgtctttagagaaactgaggcaag

8998 aagctcagctactacatgagagctggagctagtatgaaggtggaggtccagttgagtgtctttagagaaactgaggcaaG

S28ATMKO_Sg1_202

5155 aagtagactgtaatgaactggaatgagctgggccgctaagctaaactaggctggcttaaccgagatgagccaaactggaa **4nt**

AAGTAGACTGTAATGAACTGGAATGAGCTGGGCCGCtaagAGTGTGGGAACCCAGTCAAAAACCACAGAAGAGCAGGAGC

8660 cacctgggtgagcagatacagggaagctgaggcaggtaagagtgtgggaacccagtcaaaaaccacagaagagcaggagc

S28ATMKO_Sg1_212

5247 Atctaggttgaatagagctaaactctactgcctacactggactgttctgagctgagatgagctggggtgagctcagct **4nt**

ATCTAGGTTGAATAGAGCTAAACTCTACTGCCTACACTGGACAggtGGAAGTGTGGAGA**T**CCAGGCAGAGCAGCTA**C**A

8424 agaatggggatccaggtgctgcagctacaggtaagcagggacaggtggaagtgtggaga**c**ccaggcagagcagcta**t**a

S14ATMKO-Sg1-3

5265 taaactctactgcctacactggactgttctgagctgagatgagctggggtgagctcagctatgctacgcgtgttggggtg **4nt**

TAAACTCTACTGCCTACACTGGACTGTTCTGAGCTGAGATGAGGCAAGTGGGAGTGCAGAGATCCA**G**GCTGAGCAGCTCC

9030 tatgaaggtggaggtccagttgagtgtctttagagaaactgaggcaagtgggagtgcagagatcca**a**gctgagcagctcc

S14ATMKO-Sg1-8

5366 tctggagtagctgagatggggtgagatggggtgagctgagctgggctgagctggactgagctgagctagggtgagctga **4nt**

TCTGGAGTAGCTGAGATGGGGTGAGATGGGGTGAGCTGAGCAGCTATA**G**GG**C**AGCCAG**G**ACAGGTGGGAGTGTGGGGAT

8454 gtaagcagggacaggtggaagtgtggagacccaggcagagcagctata**a**gg**g**agccag**a**acaggtgggagtgtggggat

S22ATMKO_Sg1_210

5367 ctggagtagctgagatggggtgagatggggtgagctgagctgggctgagctggactgagctgagctagggtgagctgag **4nt**

ctggagtagctgagatggggtgagatggggtgagctgagctaccaaggatcagggatagacatgtaagcagtcaagctc

8925 cctggagctagtgggggtgtgggagaccaggctgagcagctaccaaggatcagggatagacatgtaagcagtcaagctc

S22ATMKO_Sg1_31

5233 aatgaacttcattaatctaggttgaatagagctaaactctactgcctacactggactgttctgagctgagatgagctggg **4nt**

AATGAACTTCATTAATCTAGGTTGAATAGAGCTAAACTCTACATGAGAGCTGGAGCTAGTATGAAGGTGGAGGTCCAGTT

8971 ggatcagggatagacatgtaagcagtcaagctcagctactacatgagagctggagctagtatgaaggtggaggtccagtt

S22ATMKO_Sg1_44

5142 ttgagccaaaatgaagtagactgtaatgaactggaatgagctgggccgctaagctaaactaggctggcttaaccgagat **4nt**

TTGAGCCAAAATGAAGTAGACTGTAATGAACTGGAATGAGCTACCAAGGATCAGGGATAGACATGTAAGCAGTCAAGCT

8924 acctggagctagtgggggtgtgggagaccaggctgagcagctaccaaggatcagggatagacatgtaagcagtcaagct

S28ATMKO_Sg1_213

5265 taaactctactgcctacactggactgttctgagctgagatgagctggggtgagctcagctatgctacgcgtgttggggtg **4nt**

taaactctactgcctacactggactgttctgagctgagatgagccaagacaactagaagtgtgtgaatccaggcagagca

8293 aaatggaagaatgcagattccaaacagaagagctacagaggagccaagacaactagaagtgtgtgaatccaggcagagca

S28ATMKO_Sg1_217

5257 aatagagctaaactctactgcctacactggactgttctgagctgagatgagctggggtgagctcagctatgctacgcgt **4nt**

aatagagctaaactctactgcctacactggactgttctgagcttagctggtataggtgacaggatggggga-attcgac

9071 aggcaagtgggagtgcagagatccaagctgagcagctccagcttagct-gtataggtgacaggatgggggata----aC

S28ATMKO_Sg1__106

5182 ctgggccgctaagctaaactaggctggcttaaccgagatgagccaaactggaatgaacttcattaatctaggttgaatag **5nt**

CTGGGCCGCTAAGCTAAACTAGGCTGGCTTAACCGAGATGAGCAAATACTACATAG**T**TGGAGCTGATGG**C**TGTATAAGGT

8821 agagaaactgatccaggtgagagtacggggtacacagctgagcaaatactacatag**c**tggagctgatgg**g**tgtataaggt

S28ATMKO_Sg1_211

5275 tgcctacactggactgttctgagctgagatgagctggggtgagctcagctatgctacgcgtgttgg **5nt**

TGCCTACACTGGACTGTTCTGAGCTGAGATGAGCTGGGGTGACAGGATGGGGGA-ATTCGACAAGG

9087 agagatccaagctgagcagctccagcttagctgtataggtgacaggatgggggata----acaagg

S14AMTKO_Sg1_106

5308 ctggggtGagctcagctat**g**ctacgc-gtgttggggtgagctgatctgaaatgagctactctggagtagctgagatgggg **5nt**

ctggggtgagctcagctat**a**ctacgctgtgttggggtgagctgg-gctgatgg**c**tgtataaggtaccaggctgagcagct

8837 gtgagagtacggggtacacagctgagcaaatactacatagctggagctgatgg**g**tgtataaggtaccaggctgagcagct

S22AMTKO_Sg1_219

5308 ctGgggtgagctcagctatgctacgc-gtgttggggtgagctgatctgaaatgagctactctggagtagctgagatgggg **5nt**

ctggggtgagctcagctatgctacgctgtgttggggtgagcaaatactacatagctggagctgatgg**c**tgtataaggtac

8823 agaaactgatccaggtgagagtacggggtacacagctgagcaaatactacatagctggagctgatgg**g**tgtataaggtac

S22AMTKO_Sg1_25

5361 g**c**tactctggagtagctgagatggggtgagatggggtgagctgagctgggctgagctggactgagctgagctagggtgag **5nt**

G**A**TACTCTGGAGTAGCTGAGATGGGGTGAGATGGGGTGAGCTGGAGCTAGTATGAAGGTGGaggtccagttgagtgtctt

8980 atagacatgtaagcagtcaagctcagctactacatgagagctggagctagtatgaaggtggaggtccagttgagtgtctT

S4ATMKO-Sg1-7

5310 gggtgagCtcagctatgctacgc-gtgttggggtgagctgatctgaaatgagctactctggagtagctgagatggggtga **6nt**

GGGTGAGCTCAGCTATG-TACGCTGTGTTGGGGTGAGCTGATCC**C**GGTGAGAGTACGGGGTACACAGCTGAGCAAATACT

8791 aagtagtagttggggattctaagcagtcacagagaaactgatcc**a**ggtgagagtacggggtacacagctgagcaaatact

S4AMTKO_Sg1_103

5308 ctggGgtgagctcagctatgctacgc-gtgttggggtgagctgatctgaaatgagctactctggagtagctgagatgggg **6nt**

ctggggtgagctcagctatgctacgctgtgttggggtgagctggagctagtatgaaggtggaggtccagttgagtgtctt

8980 atagacatgtaagcagtcaagctcagctactacatgagagctggagctagtatgaaggtggaggtccagttgagtgtctT

S22ATMKO_Sg1_211

5443 gagct-gggtgagctgagctgagctggggtgagctgagctgagctgagctgagctgagctgagctggggtgagctgag **7nt**

gagctggggtgagctgagctgagctggggtgagctgagctgatgg**c**tgtataaggtaccaggctgagcagctgaaggt

8845 acggggtacacagctgagcaaatactacatagctggagctgatgg**g**tgtataaggtaccaggctgagcagctgaaggT

S4ATMKO_Sg1_111

5388 gagatggggtgagctgagctgggctgagct**g**gactgagctgagctagggtgagctgagctgggtgagctgagctgagctg **8nt**

gagatggggtgagctgagctgggctgagct**a**gactgagctgagcaaatactacatagctggagctgatgg**c**tgtataagg

8820 cagagaaactgatccaggtgagagtacggggtacacagctgagcaaatactacatagctggagctgatgg**g**tgtataagG

S14ATMKO_Sg1_102

5281 cactggactgttctgagctgagatgagctggggtgagctcagctatgctacgcgtgttggggtgagctgatctgaaatg **10nt**

cactggactgttctgagctgagatgagctggggtgagctcagctactacatgagagctggagctagtatgaaggtggagg

8964 ctaccaaggatcagggatagacatgtaagcagtcaagctcagctactacatgagagctggagctagtatgaaggtggagg

***Aplf*^–/–^*Atm^–/–^***

APLF/ATM-DKO39_Sg1__203

MUSIGCD07 (Sμ)

5385 ggtgagatggggtgagctgagctgggctgagctggactgagctgagctagggtgagctgagctgggtgagctgagctgag **dir**

GGTGAGATGGGGTGAGCTGAGCTGGGCTGAGCTGGACTGAAGCTACCAAGGATCAGGGATAGACATGTAAGCAGTCAAGc

8922 taacctggagctagtgggggtgtgggagaccaggctgagcagctaccaaggatcagggatagacatgtaagcagtcaagc

MUSIGHANB (Sγ1)

APLF/ATM-DKO39_Sg1__211

5274 Ctgcctacactggactgttctgagctgagatgagctggggtgagctcagctatgctacgcgtgttggggtgagctgatct **dir**

CTGCCTACACTGGACTGTTCAGGGCAGCCAGGAGAAATGGAAGAATGCAGA-tccaAACAGAAGAGCTACAGAGGAGCCA

8259 gatccaggcattgtagctatagggcagccaggagaaatggaagaatgcagattccaaacagaagagctacagaggagcca

APLF/ATM-DKO70_Sg1_1

5260 agagctaaactctactgcctacactggactgttctgagctgagatgagctggggtgagctcagctatgctacgcgtgttg **dir**

AGAGCTAAACTCTACTGCCTACACTGGACTGTTCTGAGCTACAGCTGAGCAAATACTACATAGCTGGAGCTGATGGGTGT

8814 cagtcacagagaaactgatccaggtgagagtacggggtacacagctgagcaaatactacatagctggagctgatgggtgt

APLF/ATM-DKOS70_Sg1_102

5257 aataGagctaaactctactgcctacactggactgttctgagctgagatgagctggggtgagctcagctatgctacgcgtg **dir**

aatagagctaaactctactgcctacactggactgttctgccaaggatcagggatagacatgtaagcagtcaagctcagct

8928 ggagctagtgggggtgtgggagaccaggctgagcagctaccaaggatcagggatagacatgtaagcagtcaagctcagct

APLF/ATM-DKOS150_Sg1_207

5244 ttaatctaggttgaatagagctaaactctactgcctacactggactgttctgagctgagatgagctggggtgagctcag **dir**

ttaatctaggttgaatagagctaaactctactgcctacaggtaagcagggacaggtggaagtgt**g**g**a**gacccagg**c**aga

2934 aggtgggagtgtggggatccaggtctgcagctacatacgggtaagcagggacaggtggaagtgt**a**g**t**gacccagg**a**aga

APLF/ATM-DKOS222_Sg1_11

5140 gcttgagccaaaatgaagtagactgtaatgaactggaatgagctgggccgcaggttgaatagagctaaactctactgc **dir**

gcttgagccaaaatgaagtagactgtaatgaactggggagctgatgggtgtataaggtaccaggctgagcagctgaag

8844 tacggggtacacagctgagcaaataactacatagctggagctgatgggtgtataaggtaccaggctgagcagctgaag

APLF/ATM-DKOS222_Sg1_16

5221 gagccaaactggaatgaacttcattaatctaggttgaatagagctaaactctactgcctacactggactgttctgag **dir**

gagccaaactggaatgaacttcattaatctaggttgaaggattctaagcagtcacagagaaactgatccaggtgaga

8766 tgggtactcatagggaagctgggataagtagtagttggggattctaagcagtcacagagaaactgatccaggtgagA

APLF/ATM-DKO39_Sg1__224

5246 aatctaggttgaatagagctaaactctactgcctacactggactgttctgagctgagatgag **1nt**

AATCTAGGTTGAATAGAGCTAAACTCTACTGCCTACACTGGTATAGGTGACAGGATGGGGGA

9079 gggagtgcagagatccaagctgagcagctccagcttagctgtataggtgacaggatggggga

APLF/ATM-DKO70_Sg1_2

5208 Gcttaaccgagatgagccaaactggaatgaacttcattaatctaggttgaatagagctaaactctactgcctacactgga **1nt**

GCTTAACCGAGATGAGCCAAACTGGAATGAACaga-TCCAAACAGAAGAGCTACAGAGGAGCCAAGACAACTAGAAGTGT

8275 ctatagggcagccaggagaaatggaagaatgcagattccaaacagaagagctacagaggagccaagacaactagaagtgt

APLF/ATM-DKO70_Sg1_6

5463 Agctggggtgagctgagctgagctg**a**gctgagctggggtgagctgagctgagctggggtgagctgagctgagctggggt **1nt**

AGCTGGGGTGAGCTGAGCTGAGCTG**T**GCCTTAGGAGCAAGGACAGGGAAGCTATAGG**G**AAACCAGGACAGG**T**GGAAGAA

8349 aagtgtgtgaatccaggcagagcagtaccttaggagcaaggacagggaagctatagg**a**aaaccaggacagg**a**ggaagaa

APLF/ATM-DKO73_Sg1_8

5467 ggggtgagctgagctgagct**ga**gctgagct**g**gggtgagctgagctgagctggggtgagctgagctgagctggggtgagc **1nt**

GGGGTGAGCTGAGCTGAGCT**TG**GCTGAGCT**A**GGGTGAGCTGGAGCTAATTGGCACGGG**C**TGGGGTGCATGCTGG**C**TACT

8695 gtaagagtgtgggaacccagtcaaaaaccacagaagagcaggagctaattggcacggg**g**tggggtgcatgctgg**g**tact

APLF/ATM-DKO73_Sg1_9

5413 gagct**g**gactgagctgagctagggtgag**c**tgagctgggtgagctgagctgagctggggtgagctgagctgagctgagc **1nt**

GAGCT**A**GACTGAGCTGAGCTAGGGTGAG**T**TGAGCTGGGTGGTCCAGTTGAGTGTCTTTAGAGAAACTGAGGCAAGTGG

9003 cagctactacatgagagctggagctagtatgaaggtggaggtccagttgagtgtctttagagaaactgaggcaagtgg

APLF/ATM-DKO73_Sg1_12

5349 gatctgaaatgag**c**tactctggagtagctgagatggggtgagatggggtgagctgagctgggctgagctggactgagc **1nt**

GATCTGAAATGAG**A**TACTCTGGAGTAGCTGAGATGGGGTGaa-TAGTAGTTGGGGATTCTAAGCAGTCACAGAGAA**G**C

8751 gggtggggtgcatgctgggtactcatagggaagctgggataagtagtagttggggattctaagcagtcacagagaa**a**c

APLF/ATM-DKO73_Sg1_13

5145 agccaaaatgaagtagactgtaatgaactggaat**g**agctgggccgctaagctaaactaggctggct **1nt**

AGCCAAAATGAAGTAGACTGTAATGAACTGGAAT**T**AGCTGGTATAGGTGACAGGATGGGGGA-A

9079 gggagtgcagagatccaagctgagcagctccagcttagctgtataggtgacaggatgggggata

APLF/ATM-DKO73_Sg1_25

5410 gctgagct**g**gactgagctgagctagggtgagctgagctgggtgagctgagctgagctggggtgagctgagctgagctg **1nt**

GCTGAGCT**A**GACTGAGCTGAGCTAGGGTGAGCTGAGCTGGAAGAGCAGGAGCTAATTGGCACGGG**C**TGGGGTGCATGC

8688 gaggcaggtaagagtgtgggaacccagtcaaaaaccacagaagagcaggagctaattggcacggg**g**tggggtgcatgc

APLF/ATM-DKOS70_Sg1_122

5169 Gaactggaatgagctgggccgctaagctaaactaggctggcttaaccgagatgagccaaactggaatgaacttcattaat **1nt**

gaactggaatgagctgggccgctaagctaaactaggctggctgatcc**c**ggtgagagtacggggtacacagctgagcaaat

8788 gataagtagtagttggggattctaagcagtcacagagaaactgatcc**a**ggtgagagtacggggtacacagctgagcaaat

APLF/ATM-DKOS73_Sg1_120

5220 tgagccaaactggaatgaacttcattaatctaggttgaatagagctaaactctactgcctacactggactgttctgagc **1nt**

tgagccaaactggaatgaacttcattaatctaggttgaaccaaggatcagggatagacatgtaagcagtcaagctcagc

8928 ggagctagtgggggtgtgggagaccaggctgagcagctaccaaggatcagggatagacatgtaagcagtcaagctcagc

APLF/ATM-DKOS222_Sg1_12

5366 tctggagtagctgagatggggtgagatggggtgagctgagctgggctgagctggactgagctgagctagggtgagct **1nt**

tctggagtagctgagatggggtgagatggggtgagctgagagtgtggggatccaggtaaggc**a**ggactggggag**t**ca

8482 acccaggcagagcagctataagggagccagaacaggtgggagtgtggggatccaggtaaggc**t**ggactggggag**c**ca

APLF/ATM-DKOS222_Sg1_17

5413 gagctggactgagctgagctagggtgagctgagctgggtgagctgagctgagctggggtgagctgagctgagctgag **1nt**

gagctggactgagctgagctagggtgagctgagctgggtaagcagtcacagagaaactgatccaggtgagagtacgg

8772 ctcatagggaagctgggataagtagtagttggggattctaagcagtcacagagaaactgatccaggtgagagtacgG

APLF/ATM-DKO-S39_Sg1__120

5169 Tgaactggaatgagctgggccgctaagctaaactaggctggcttaaccgagatgagccaaactggaatgaacttcatta **2nt**

TGAACTGGAATGAGCTGGGCCGCTAAGCTAAACTAGGCTGGACAGGTGGAAGTGTGGTGACCCAGGCAGAGCAGCTATA

7882 aagtgtggtgacccaggcagagaagctccagggcagccaggacaggtggaaatgtggtgacccaggcagagtagctata

APLF/ATM-DKO39_Sg1__206

5253 gttgaatagag**c**taaactctactgcctacactggactgttctgagctgagatgagctggggtgagctcagctatgctacg **2nt**

GTTGAATAGAG**G**TAAACTCTACTGCCTACACTGGACTgttcATAGGGAAGCTGGGATAAGTAGTAGTTGGGGATTCTAAG

8734 aggagctaattggcacggggtggggtgcatgctgggtactcatagggaagctgggataagtagtagttggggattctaag

APLF/ATM-DKO70_Sg1_12

5260 agagctaaactctactgcctaca**c**tggactgttctgagctgagatgagctggggtgagctcagctatgctacgcgtgt **2nt**

AGAGCTAAACTCTACTGCCTACA**G**TGGACTGTTCTGAGCAGAGCAGCTATA**G**GG**C**AGCCAG**G**ACAGGTGGGAGTGTGG

8451 caggtaagcagggacaggtggaagtgtggagacccaggcagagcagctata**a**gg**g**agccag**a**acaggtgggagtgtgg

APLF/ATM-DKO150-Sg1-6

5509 gctgagctggggtgagctgagctgagctggggt**g**agctgagctgagct **2nt**

GCTGAGCTGGGGTGAGCTGAGCTGAGCTGGGGT**A**AGCTGG**G**ACGGG**C**TGGGGTGCATGCTGG**C**TACTCATAGGGAAGC

8707 gaacccagtcaaaaaccacagaagagcaggagctaatTgg**c**acggg**g**tggggtgcatgctgg**g**tactcatagggaagc

APLF/ATM-DKOS70_Sg1_103

5326 tgctacgc-gtgttggggtgagctgatctgaaatgagctactctggagtagctgagatggggtgagatggggtgagctga **2nt**

tgctacgctgtgttggggtgagctgatctgaaatgagctaaggtaccaggctgagcagctgaaggtaacctggagctagt

8857 gctgagcaaatactacatagctggagctgatgggtgtataaggtaccaggctgagcagctgaaggtaacctggagctagt

APLF/ATM-DKOS70_Sg1_105

5170 aactggaatgagctgggccgctaagctaaactaggctggcttaaccgagatgagccaaactggaatgaacttcattaatc **2nt**

aactggaatgagctgggccgctaagctaaactaggctggctgatcc**c**ggtgagagtacggggtacacagctgagcaaata

8789 ataagtagtagttggggattctaagcagtcacagagaaactgatcc**a**ggtgagagtacggggtacacagctgagcaaata

APLF/ATM-DKOS70_Sg1_107

8315 Gagctcagctatgctacgc-gtgttggggtgagctgatctgaaatgagctactctggagtagctgagatggggtgagatg **2nt**

gagctcagctatgctacgctgtgttggggtgagctgatctgggagacc**t**ggctgagcagctaccaaggatcagggataga

8905 ggctgagcagctgaaggtaacctggagctagtgggggtgtgggagacc**a**ggctgagcagctaccaaggatcagggataga

APLF/ATM-DKOS70_Sg1_118

5271 ctactgcctacactggactgttctgagctgagatgagctggggtgagctcagctatgctacgcgtgttggggtgagctga **2nt**

ctactgcctacactggactgttctgagctgagatgagctggtgggagtgtggggatccaggtaaggctggactggggagc

8477 tggagacccaggcagagcagctataagggagccagaacaggtgggagtgtggggatccaggtaaggctggactggggagc

APLF/ATM-DKOS222_Sg1_9

5293 ctgagctgagatgagctggggtgagctcagctatgctacgcgtgttggggtgagctgatctgaaatgagctactctgg **2nt**

ctgagctgagatgagctggggtgagctcagctatgctacgctgaaggtaacctggagctagtgggggtgtgggagacc

8875 agctggagctgatgggtgtataaggtaccaggctgagcagctgaaggtaacctggagctagtgggggtgtgggagacc

APLF/ATM-DKOS222_Sg1_10

5400 gctgagctgggctgagct**g**gactgagctgagctagggtgagctgagctgggtgagctgagctgagctggggtgagctg **2nt**

gctgagctgggctgagct**a**gactgagctgagctagggtgagggaagctgggataagtagtagttggggattctaagca

8738 gctaattggcacggggtggggtgcatgctgggtactcatagggaagctgggataagtagtagttggggattctaagca

APLF/ATM-DKOS222_Sg1_14

5179 gagctgggccgctaagctaaactaggctggcttaaccgagatgagccaaactggaatgaacttcattaatctaggtt **2nt**

gagctgggccgctaagctaaactaggctggcttaaccgatcc**c**ggtgagagtacggggtacacagctgagcaaatac

8793 gtagtagttggggattctaagcagtcacagagaaactgatcc**a**ggtgagagtacggggtacacagctgagcaaatac

APLF/ATM-DKOS222_Sg1_19

5407 tgggctgagctggactgagctgagctagggtgagctgagctgggtgagctgagctgagctggggtgagctgagctga **2nt**

tgggctgagctggactgagctgagctagggtgagctgaggtggaagtgtggagacccaggcagagcagctataaggg

8429 ggggatccaggtgctgcagctacaggtaagcagggacaggtggaagtgtggagacccaggcagagcagctataaggg

APLF/ATM-DKO-Sg1-4

5169 Gaactggaatga**g**ctgggccgctaagctaaactaggctggcttaaccgagatgagccaaactggaatgaacttcattaat **2nt**

GAACTGGAATGA**A**CTGGGCCGCTAAGCTAAACTAGGCTGGCTGATCC**C**GGTGAGAGTACGGGGTACACAGCTGAGCAAAT

8789 gataagtagtagttggggattctaagcagtcacagagaaactgatcc**a**ggtgagagtacggggtacacagctgagcaaat

APLF/ATM-DKO-Sg1-17

5174 ggaatgagctgggccgctaagctaaactaggctggcttaaccgagatgagccaaactggaatgaaccttcattaatctag **2nt**

GGAATGAGCTGGGCCGCTAAGCTAAACTAGGCTGGCTTAACCAGGACAGGTGGAAGTGTGGTGACCCAGGCAGAG**C**AGCT

7878 ggtggaagtgtggtgacccaggcagagcagctatagggagccaggacaggtggaagtgtggtgacccaggcagag**a**agcT

APLF/ATM-DKOS39_Sg1__101

5387 tgagatGgggtgagctgagctgggctgagctggactgagctgagctagggtgagctgagctgggtgagctgagctgagc **3nt**

tgagatGGGGTGAGCTGAGCTGGGCTGAGCTGGACTGAGCTGGAGCTAGTGGGGGTGTGGGAGACCAGGCTGAGCAGCT

8887 tgggtgtataaggtaccaggctgagcagctgaaggtaacctggagctagtgggggtgtgggagaccaggctgagcagct

APLF/ATM-DKO70_Sg1_3

5140 Gcttgagccaaaatgaagtagactgtaatgaactggaatgagctgggccgctaagctaaactaggctggcttaaccgag **3nt**

GCTTGAGCCAAAATGAAGTAGACTGTAATGAACAGAAGAGCTACAGAGGAGCCAAGACAACTAGAAGTGTGTGAATCCA

8285 gccaggagaaatggaagaatgcagattccaaacagaagagctacagaggagccaagacaactagaagtgtgtgaatcca

APLF/ATM-DKO73_Sg1_1

5164 Gtaatgaactggaatgagctgggccgctaagctaaactaggctggcttaaccgagatgagccaaactggaatgaactt **3nt**

GTAATGAACTGGAATGAGCTGGGCCGCTAAGCTAAACTAGCTGGAGCTGATGG**C**TGTATAAGGTACCAGGCTGAGCAG

8837 gtgagagtacggggtacacagctgagcaaatactacatagctggagctgatgg**g**tgtataaggtaccaggctgagcag

APLF/ATM-DKO73_Sg1_7

5178 tgagctgggccgctaagctaaactaggctggcttaaccgagatgagccaaactggaatgaacttcattaatctaggttg **3nt**

TGAGCTGGGCCGCTAAGCTAAACTAGGCTGGCTTAACCGAGAATGGGGATCCAGGTGCTGCAGCTACAGGTAAGCAGGG

8385 caaggacagggaagctataggaaaaccaggacaggaggaagaatggggatccaggtgctgcagctacaggtaagcaggg

APLF/ATM-DKO73_Sg1_36

5277 Ctacactggactgttctgagctgagatgagctggggtgagctcagctatgctacgcgtgttggggtgagctgatctga **3nt**

CTACACTGGACTGTTCTGAGCTGAGATgCTGG**C**TACTCATAGGGAAG**T**TGGGATAAGTAGTAGTTGGGGATTCTAAGc

8737 agctaattggcacggggtggggtgcatgctgg**g**tactcatagggaag**c**tgggataagtagtagttggggattctaagc

APLF/ATM-DKOS70_Sg1_120

5167 atgaactggaatgagctgggccgctaagctaaactaggctggcttaaccgagatgagccaaactggaatgaacttcatta **3nt**

atgaactggaatgagctgggccgctaagctaaactaggctggggtgcatgctgg**c**tactcatagggaagctgggataagt

8715 tcaaaaaccacagaagagcaggagctaattggcacggggtggggtgcatgctgg**g**tactcatagggaagctgggataagt

APLF/ATM-DKOS73_Sg1_108

5179 gagctgggccgctaagctaaactaggctggcttaaccgagatgagccaaactggaatgaacttcattaatctaggttga **3nt**

gagctgggccgctaagctaaactaggctggcttaaccgagatcc**c**ggtgagagtacggggtacacagctgagcaaatac

8791 aagtagtagttggggattctaagcagtcacagagaaactgatcc**a**ggtgagagtacggggtacacagctgagcaaatac

APLF/ATM-DKOS73_Sg1_109

5283 actggactgttctgagctgagatgagctggggtgagctcagctatgctacgcgtgttggggtgagctgatctgaaatga **3nt**

actggactgttctgagctgagatgagctggggtgagctcagcagtcacagagaa**g**ctgatcc**c**ggtgagagtacggggt

8773 tcatagggaagctgggataagtagtagttggggattctaagcagtcacagagaa**a**ctgatcc**a**ggtgagagtacggggt

APLF/ATM-DKOS150_Sg1_201

5185 ggccgctaagctaaactaggctggcttaaccgagatgagccaaactggaatgaacttcattaatctaggttgaatagag **3nt**

ggccgctaagctaaactaggctggcttaaccgagatgagctcagctactacatgagagctggagctagtatgaaggtgg

8962 agctaccaaggatcagggatagacatgtaagcagtcaagctcagctactacatgagagctggagctagtatgaaggtgg

APLF/ATM-DKOS222_Sg1_21

5256 gaatagagctaaactctactgcctacactggactgttctgagctgagatgagctggggtgagctcagctatgctacg **3nt**

gaatagagctaaactctactgcctacactggactgttctgggtgagcagatacagggaagctgaggcaggtaagagt

8626 gttgtagaggaacaggggcagattagaatgaatgcacctgggtgagcagatacagggaagctgaggcaggtaagagT

APLF/ATM-DKO39_Sg1__208

5315 gagctCagctatgctacgc-gtgttggggtgagctgatctgaaatgagctactctggagtagctgagatggggtgagatg **4nt**

GAGCTCAGCTATGCTACGCTGTGTTGGGGTGAGCTGATCTGAGCAGCTACCAAGGATCAGGGATAGACATGTAAGCAGTC

8918 aaggtaacctggagctagtgggggtgtgggagaccaggctgagcagctaccaaggatcagggatagacatgtaagcagtc

APLF/ATM-DKO39_Sg1__214

5277 cctacactggactgttctgagctgagatgagctggggtgagctcagctatgctacgcgtgttggggtgagctgatctga **4nt**

CCTACACTGGACTGTTCTGAGCTGAGATGAGCTGGGGTGAGGCAAGTGGGAGTGCAGAGATCCAAGCTGAGCAGCTCCA

9032 tgaaggtggaggtccagttgagtgtctttagagaaactgaggcaagtgggagtgcagagatccaagctgagcagctcca

APLF/ATM-DKO39_Sg1__220

5394 gggtgagctgagctgggctgagctggactgagctgagctagggtgagctgagctgggtgagctgagctgagctggggtg **4nt**

GGGTGAGCTGAGCTGGGCTGAGCTGGACTGAGCTGAGCTAGGTGGAATGTGAATATCCAGGCAGAACAGGTCCAGGGTG

8524 tgtggggatccaggtaaggctggactggggagccaaggtaggtggaatgtgaatatccaggcagaacaggtccagggtg

APLF/ATM-DKO73_Sg1_28

5390 gatggggtgagctgagctgggctgagct**g**gactgagctgagctagggtgagctgagctgggtgagctgagctgagctg **4nt**

GATGGGGTGAGCTGAGCTGGGCTGAGCT**A**GACTGAGCTGAGCagcTATA**G**GG**C**AGCCAG**G**ACAGGTGGGAGTGTGGGG

8453 ggtaagcagggacaggtggaagtgtggagacccaggcagagcagctata**a**gg**g**agccag**a**acaggtgggagtgtgggg

APLF/ATM-DKO73_Sg1_37

5487 tgagatggggtgagctgagctgggctgagct**g**gactgagctgagctagggtgagctgagctgggtgagctgagctgag **4nt**

TGAGATGGGGTGAGCTGAGCTGGGCTGAGCT**A**GACTGAGCTATA**G**GG**C**AGCCAG**G**ACAGGTGGGAGTGTGGGGATCca

8458 gcagggacaggtggaagtgtggagacccaggcagagcagctata**a**gg**g**agccag**a**acaggtgggagtgtggggatcca

APLF/ATM-DKO150-Sg1-2

5224 ccaaactggaatgaacttcattaatctaggttgaatagagctaaactctactgcctacactggactgttctgagctga **4nt**

CCAAACTGGAATGAACTTCATTAATCTAGGTTGAATAGAGCAAATACTACATAGCTGGAGCTGATGG**C**TGTATAAGGT

8823 agaaactgatccaggtgagagtacggggtacacagctgagcaaatactacatagctggagctgatgg**g**tgtataaggt

APLF/ATM-DKO150-Sg1-13

5229 Ctggaatgaacttcattaatctaggttgaatagagctaaactctactgcctacactggactgttctgagctgagatga **4nt**

CTGGAATGAACTTCATTAATCTAGGTTGAATAGAGCTAAACCAGGACAGG**T**GGAAGAATGGGGATCCAGGTGCTGCAG

8370 gcagtaccttaggagcaaggacagggaagctataggaaaaccaggacagg**a**ggaagaatggggatccaggtgctgcag

APLF/ATM-DKOS73_Sg1_113

5236 gaacttcattaatctaggttgaatagagctaaactctactgcctacactggactgttctgagctgagatgagctggg **4nt**

gaacttcattaatctaggttgaatagagctaaactctactgaggcaagtgggagtgcagagatcca**g**gctgagcagctc

9029 tatgaaggtggaggtccagttgagtgtctttagagaaactgaggcaagtgggagtgcagagatcca**a**gctgagcagctc

APLF/ATM-DKOS150_Sg1_206

5402 tgagctgggctgagct**g**gactgagctgagctagggtgagctgagctgggtgagctgagctgagctggggtgagctgagc **4nt**

tgagctgggctgagct**a**gactgagctgagctagggtgagctcagctactacatgagagctggagctagtatgaaggtgg

8962 agctaccaaggatcagggatagacatgtaagcagtcaagctcagctactacatgagagctggagctagtatgaaggtgg

APLF/ATMDKOS222_Sg1_5

5374 agctgagatggggtgagatggggtgagctgagctgggctgagctggactgagctgagctagggtgagctgagctgggt **4nt**

agctgagatggggtgagatggggtgagctgagctgggctgatcc**c**ggtgagagtacggggtacacagctgagcaaata

8791 aagtagtagttggggattctaagcagtcacagagaaactgatcc**a**ggtgagagtacggggtacacagctgagcaaata

APLF/ATM-DKOS222_Sg1_8

5166 aatgaactggaatgagctgggccgctaagctaaactaggctggcttaaccgagatgagccaaactggaatgaacttca **4nt**

aatgaactggaatgagctgggccgctaagctaaactaggctggagctgatgg**c**tgtataaggtaccaggctgagcagc

8838 tgagagtacggggtacacagctgagcaaatactacatagctggagctgatgg**g**tgtataaggtaccaggctgagcagC

APLF/ATM-DKOS73_Sg1_104

5399 agctgagctgggctgagct**g**gactgagctgagctagggtgagctgagctgggtgagctgagctgagctggggtgagctg **5nt**

agctgagctgggctgagct**a**gactgagctgagctagggtgagcagctgaaggtaacctgga**c**ctagtgggggtgtggga

8870 tacatagctggagctgatgggtgtataaggtaccaggctgagcagctgaaggtaacctgga**g**ctagtgggggtgtggga

APLF/ATM-DKOS73_Sg1_110

5300 gagatgaGctggggtgagctcagctatgctacgc-gtgttggggtgagctgatctgaaatgagctactctggagtagct **5nt**

gagatgagctggggtgagctcagctatgctacgctgtgttgggggtgtgggagacc**t**ggctgagcagctaccaaggatc

8897 aggtaccaggctgagcagctgaaggtaacctggagctagtgggggtgtgggagacc**a**ggctgagcagctaccaaggatC

APFL/ATM-DKO73_Sg1_6

5255 tgaatagagctaaactctactgcctacactggactgttctgagctgagatgagctggggtgagctcagctatgctacgcg **6nt**

TGAATAGAGCTAAACTCTACTGCCTACACTGGACTGTTCTGAGCAGCTCCAGCTTAGCTGGTATAGGTGACAGGATGGGG

9060 tagagaaactgaggcaagtgggagtgcagagatccaagctgagcagctccagcttagct-gtataggtgacaggatgggg

APLF/ATM-DKOS70_Sg1_108

5189 gctaagctaaactaggctggcttaaccgagatgagccaaactggaatgaacttcattaatctaggttgaatagagctaaa **6nt**

gctaagctaaactaggctggcttaaccgagatgagccaaactgaggcaagtgggagtgcagagatcca**g**gctgagcagct

9028 agtatgaaggtggaggtccagttgagtgtctttagagaaactgaggcaagtgggagtgcagagatcca**a**gctgagcagcT

APLF/ATM-DKOS70_Sg1_116

5268 Actctactgcctacact**g**gactgttctgagctgagatgagctggggtgagctcagctatgctacgcgtgttggggtgagc **6nt**

actctactgcctacact**a**gactgttctgagctgagatgagctgatgg**c**tgtataaggtaccaggctgagcagctgaaggt

8843 gtacggggtacacagctgagcaaatactacatagctggagctgatgg**g**tgtataaggtaccaggctgagcagctgaaggt

APLF/ATM-DKOS222_Sg1_2

5346 gctgatctgaaatgag**c**tactctggagtagctgagatggggtgagatggggtgagctgagctgggctgagctggactg **6nt**

gctgatctgaaatgag**a**tactctggagtagctgagatggggtggggtgcatgctgg**c**tactcatagggaagctgggat

8713 agtcaaaaaccacagaagagcaggagctaattggcacggggtggggtgcatgctgg**g**tactcatagggaagctgggat

APLF/ATM-DKOS222_Sg1_4

5367 ctggagtagctgagatggggtgagatggggtgagctgagctgggctgagctggactgagctgagctagggtgagctg **6nt**

ctggagtagctgagatggggtgagatggggtgagctgagctgatgggtgtataaggtaccaggctgagcagctgaag

8844 tacggggtacacagctgagcaaatactacatagctggagctgatgggtgtataaggtaccaggctgagcagctgaaG

APLF/ATM-DKO73_Sg1_11

5456 tgagct**g**agctggggtgagctgagctgagct**ga**gctgagctggggtgagctgagctgagctggggtgagctgagctga **7nt**

TGAGCT**A**AGCTGGGGTGAGCTGAGCTGAGCT**TG**GCTGAGCTGGAGCTAGTATGAAGGTGGAGGTCCAGTTGAGTGTCt

8981 tagacatgtaagcagtcaagctcagctactacatgagagctggagctagtatgaaggtggaggtccagttgagtgtct

APLF/ATM-DKOS73_Sg1_101

5373 tagctgagatggggtgagatggggtgagctgagctgggctgagctggactgagctgagctagggtgagctgagctgggt **7nt**

tagctgagatggggtgagatggggtgagctgagctgggctgagcagctcca**a**cttagctggtataggtgacaggatggg

9060 tagagaaactgaggcaagtgggagtgcagagatccaagctgagcagctcca**g**cttagct-gtataggtgacaggatggG

APLF/ATM-DKOS222_Sg1_3

5438 gagctgagctgggtgagctgagct**g**agctggggtgagctgagctgagctgagctgagctggggtgagctgagctgagc **8nt**

gagctgagctgggtgagctgagct**a**agctggggtgagctgagcagctccagcttagctggtataggtgacagga**g**ggg

9061 agagaaactgaggcaagtgggagtgcagagatccaagctgagcagctccagcttagct-gtataggtgacagga**t**ggg

**Figure S4**

**
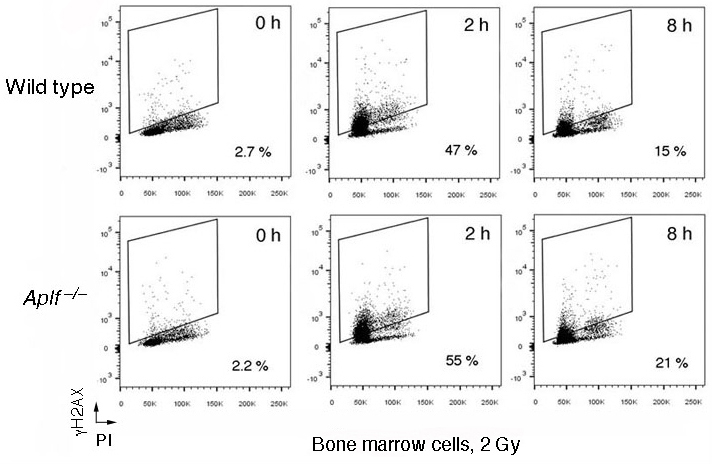
**

**Figure S5**

**
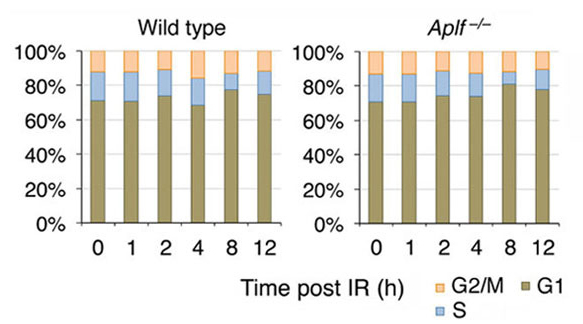
**

**Figure S6**

**
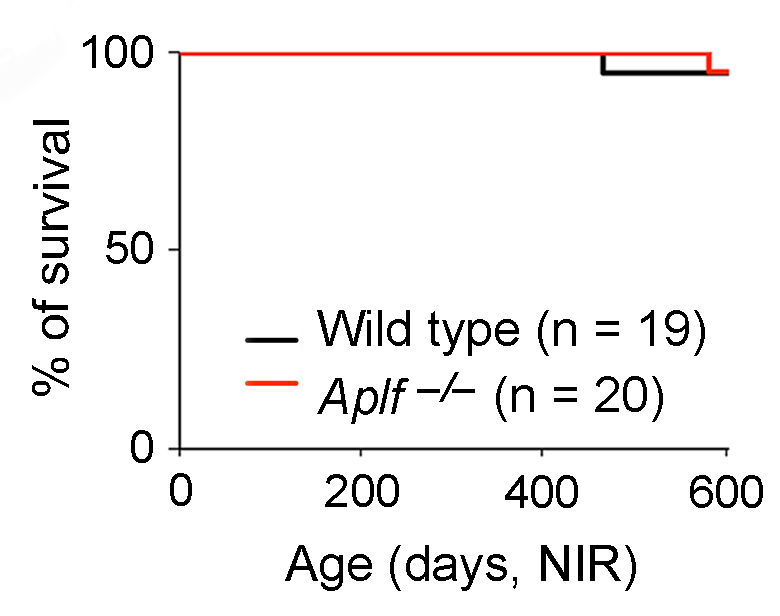
**

**Figure S7**

**
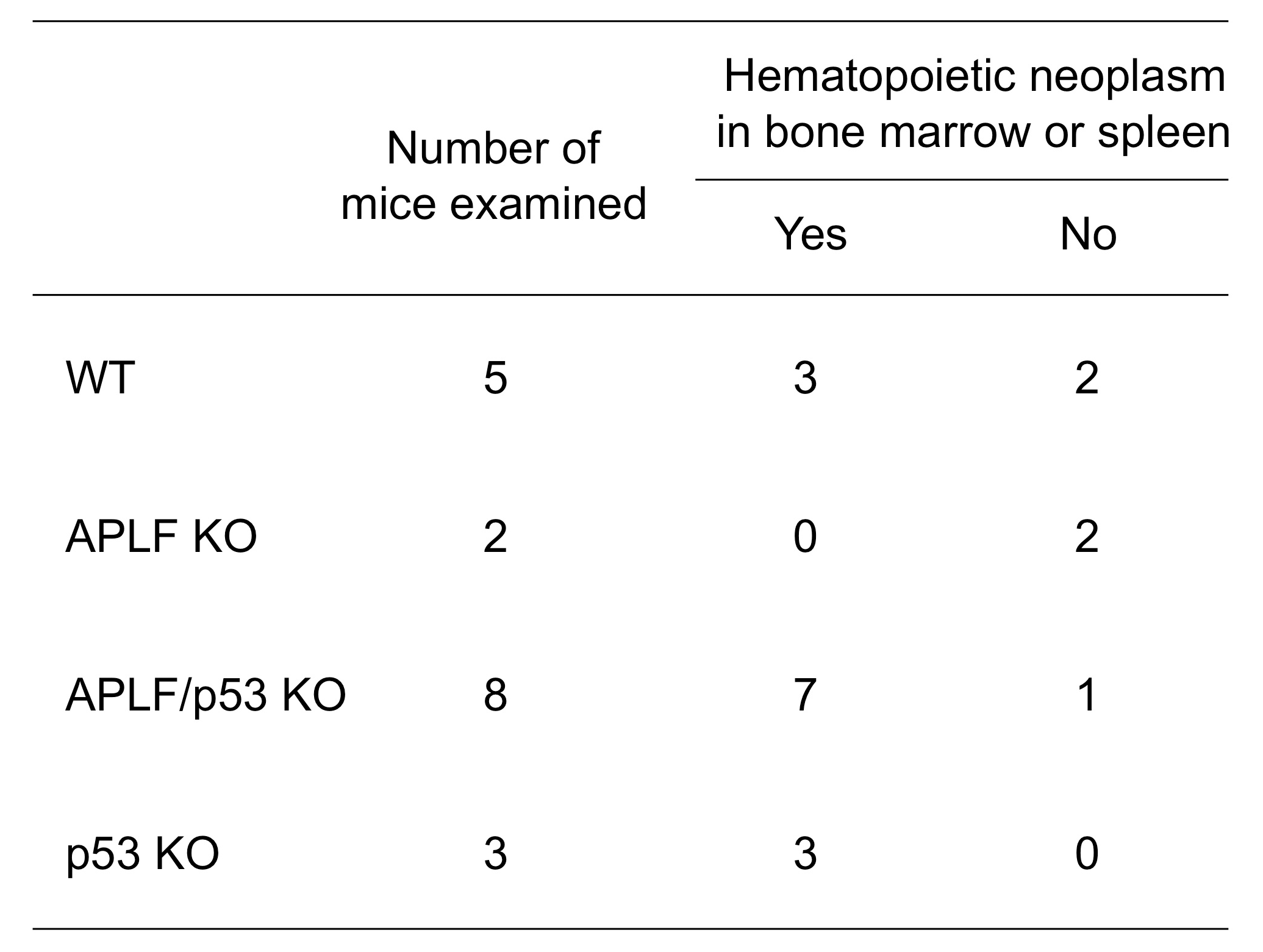
**

**Figure S8**


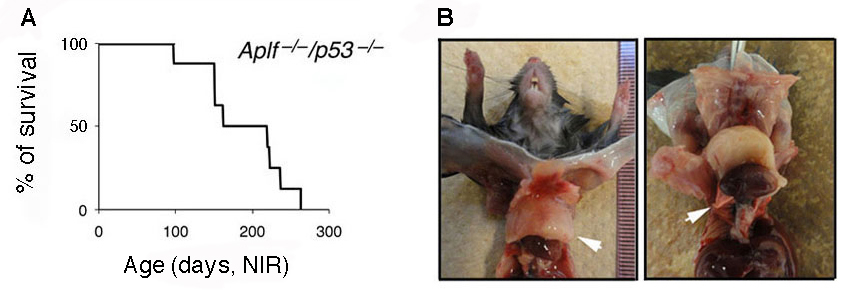


**Figure S9**


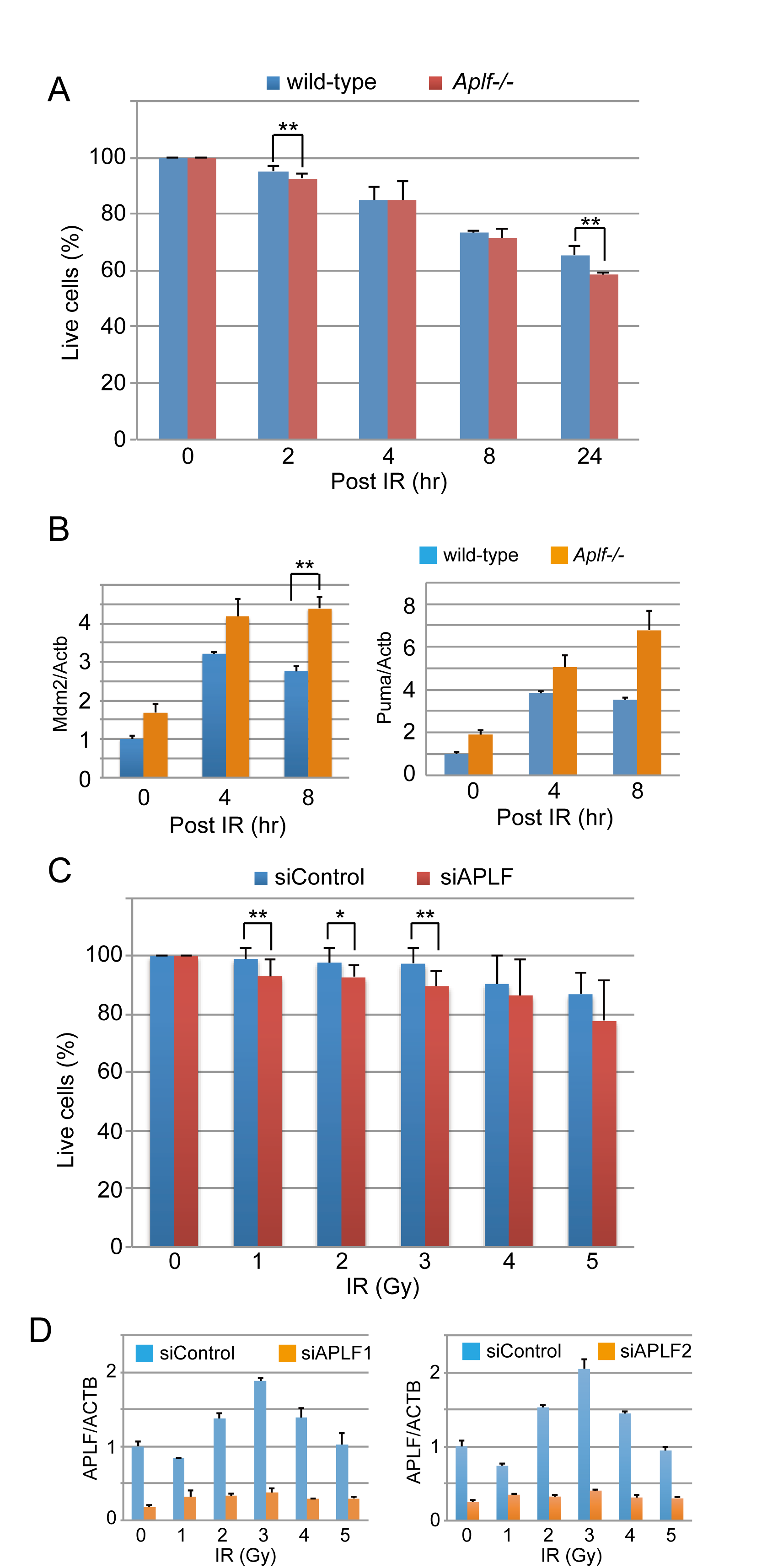


**Figure S10**

|  | **γH2Ax positive bone marrow cells (%)** | | | | | | | | | |
| --- | --- | --- | --- | --- | --- | --- | --- | --- | --- | --- |
| **Time post 2Gy** | **WT 1** | **WT 2** | **WT 3** | **Mean** | **SD** | **KO 1** | **KO 2** | **KO 3** | **Mean** | **SD** |
| C | 2.7 | 2.72 | 2.41 | 2.6 | 0.17 | 2.21 | 2.38 | 2.11 | 2.2 | 0.14 |
| 0.5 h | 75 | 75.2 | 73.9 | 74.7 | 0.70 | 76.9 | 77.3 | 77 | 77.1 | 0.21 |
| 1 h | 71.9 | 71.3 | 70.9 | 71.4 | 0.50 | 78.2 | 77.3 | 76.9 | 77.5 | 0.67 |
| 2 h | 47.1 | 45.4 | 45.1 | 45.9 | 1.07 | 55.4 | 55.4 | 54.5 | 55.1 | 0.52 |
| 4 h | 42.1 | 42.3 | 41.1 | 41.8 | 0.64 | 46.2 | 46 | 45 | 45.7 | 0.64 |
| 6 h | 23.8 | 23.3 | 23.3 | 23.5 | 0.29 | 28.4 | 28 | 27.3 | 27.9 | 0.56 |
| 8 h | 14.9 | 15.2 | 15.3 | 15.1 | 0.21 | 20.6 | 20.6 | 19.9 | 20.4 | 0.40 |

SD = standard deviation

**Figure S11**

**
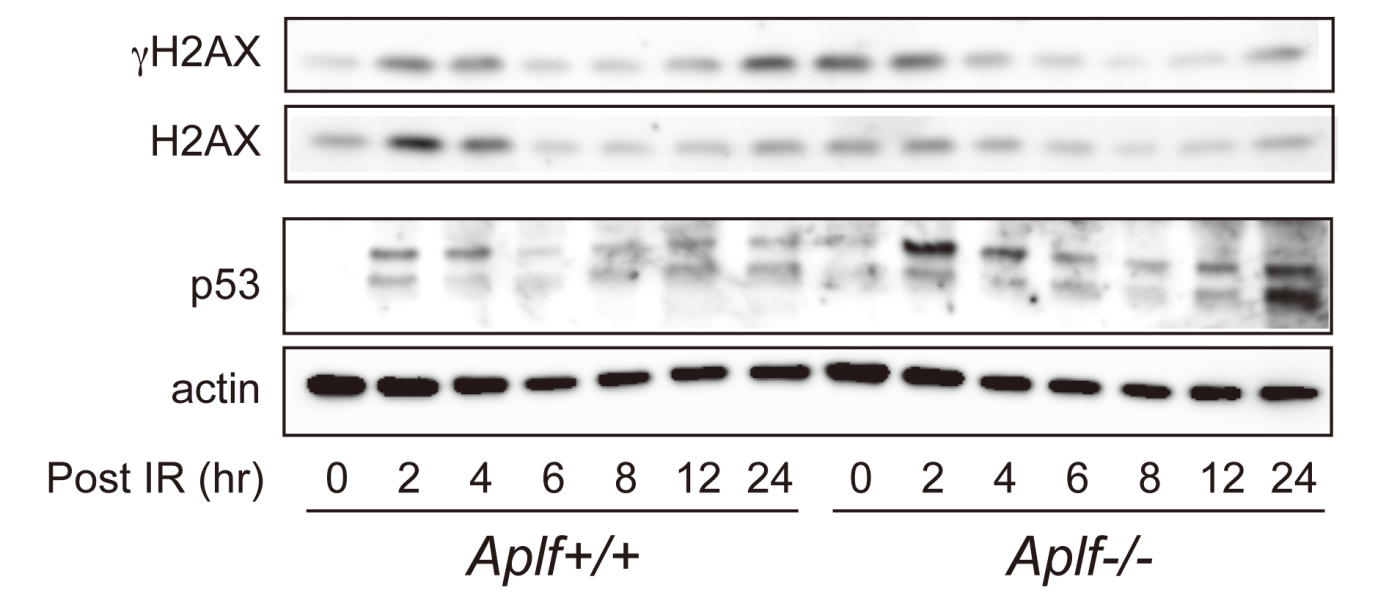
**

**Supplementary Figure Legends**

**Figure S1: Gene targeting of *Aplf.***

(A) Gene targeting strategy for creating *Aplf^–/–^* mouse and protein architecture of APLF. Schematic diagram of protein structure of APLF (top panel), the wild type *Aplf* locus (second), the targeting construct (third), and the mutated *Aplf* allele (bottom). The coding exons are shown as black boxes. Exons 2 and 3 were replaced by *PGKneo* (Neo) cassette in opposite transcriptional direction to that of *Aplf*. The 5’ flanking probe 1 used for the Southern blot analysis in B is as indicated by black bar, with positions and the predicted fragment sizes for detecting wild type and mutant alleles depicted by arrows. Probe 2, in black bar, hybridizes the neomycin gene in mutant allele. Arrowheads denote the primer pairs for genotyping (a, b; a, c). Grey box in top panel represents Forkhead associated domain (FHA), which is encoded by exons 1 – 3. Open boxes are the two DNA binding PBZ motifs. B, *Bam*HI; N, *Not*I; S, *Sal*I; X, *Xho*I; Xb, *Xba*I. (B) Southern blot confirmation of positively targeted mutant ES cells. Numbers for each tested ES cell clones are shown on top of each lane. (C) Semi-quantitative RT-PCR showing expression of *Aplf* and *β-Actin* in (1) ear, (2) liver, (3) brain, (4) lung, (5) thymus, (6) splenic B cells, (7) peritoneal B cells of wild type mouse, and (8) splenic B cells, (9) peritoneal B cells of *Aplf^–/–^* mouse.

**Figure S2: B and T cell development, and immune response in *Aplf^–/–^* mice.**

(A) Representative flow cytometric analysis of the differentiated pro-pre B cells and immature B cells in bone marrow; IgM^+^ B cells in spleens; double positive (CD4^+^CD8^+^), single positive (CD4^+^ or CD8^+^), and αβTCR^+^ maturing T cells in thymus of 5 weeks old mice with genotypes as indicated. (B) Average total thymocytes and average CD4^+^CD8^+^ T cells in thymus of the indicated genotypes. Data are mean ± s.d. *P* values were determined by unpaired two-tailed Student’s *t*-test. (C) Average total splenocytes and average IgM^+^ B cells in spleens of the indicated genotypes. Data are mean ± s.d. *P* values were determined by unpaired two-tailed Student’s *t*-test. (D) Class switching to IgG1 of indicated genotypes. Images were representative of three independent experiments. (E) Percentage of IgG1. Data shown are the mean ± SD of 3 – 4 mice of each group. *P* value was obtained by unpaired Student’s *t*-test.

**Figure S3:** **Sμ/Sγ1 switch junction analysis.**

Sμ/Sγ1 CSR in the CD43^–^ splenic B cells of wild type, *Aplf^–/–^*, *Atm^–/–^*, *Aplf^–/–^Atm^–/–^* mice stimulated by IL4 and LPS for 4 days. Sμ/Sγ1 switch junctions were analysed by aligning against Genbank files MUSIGCD07 for Sμ and MUSIGHANB for Sγ1. The nucleotide numbers according to the Genbank files of the Sμ and Sγ1 sequence are indicated to the left of the top (black, Sμ) and bottom (red, Sγ1) sequences of each alignment. The middle sequences are sequenced data of clones with ID as denoted before each alignment and colours matching to homologous sequences of either Sμ (top, black) or Sγ1 (bottom, red). In all junctions analysed, no gaps or insertions directly at the Sμ/Sγ1 breakpoints were allowed. Gray boxes shaded the overlapping nucleotides (nt) in blue. Mutations are boldfaced and deletions are represented by a dash. Numbers of overlapping nucleotides (homology) are indicated to the right of each alignment boldfaced in black. DIR, direct joins.

**Figure S4: Quantification of γH2AX positive cells after IR exposure.**

Raw flow cytometry data quantifying cells with γH2AX positive signals after IR exposure. A representative of three independent experiments is shown. PI, propidium iodide.

**Figure S5: Comparable cell cycle distribution between WT and *Aplf*^–/–^ mice after IR exposure.**

Spleen cells were exposed to 2 Gy IR and cell cycle phases were evaluated by measuring propidium iodide-stained DNA content. G1, S, and G2/M phases were defined as 2N = G1, 2N < S < 4N, and 4N = G2/M. Data shown are average of four biological replicates.

**Figure S6: Comparable life span between Wild type and *Aplf*^–/–^ mice without irradiation treatment.**

Kaplan-Meier survival curve of non-irradiated (NIR) wild type and *Aplf^–/–^* mice of C57BL/6J background.

**Figure S7: Frequency of hematopoietic neoplasms in irradiated mice.**

**Figure S8: Thirty percent of irradiated *Aplf^–/–^p53^–/–^* mice developed and died from thymic lymphoma.**

(A) Kaplan-Meier survival curve of non-irradiated (NIR) *Aplf^–/–^p53^–/–^* mice (n = 8). (B) A representative case of thymic lymphoma (arrowhead, left) found in the irradiated *Aplf^–/–^p53^–/–^* mice. Mice died due to the collapse of the lungs (arrowhead, right).

**Figure S9: Enhanced IR induced apoptosis in *Aplf^-/-^* bone marrow and APLF depleted MCF10A cells.**

(A) Apoptosis in wild type and *Aplf^–/–^* bone marrow cells after IR exposure (n = 4) was evaluated by staining cells with AnnexinV-FITC and PI. Fluorescence positive cells were quantified by flow analysis using (BD FACSCalibur) and analyzed by FlowJo. (B) Relative gene expression of p53 target genes, *mdm2* and *puma*, against *β-actin* (Actb) control in wild type and *Aplf^–/–^* bone marrow cells after 2Gy of IR exposure (n = 3) was evaluated by quantitative real-time PCR (qRT-PCR) and analyzed by 2^–ΔΔCt^ method. (C) Apoptosis in MCF10A cells depleted for APLF after IR exposure (n = 4) was quantified as described in (A). (D) Relative APLF transcript levels in MCF10A cells depleted for APLF after IR exposure (n = 4) were evaluated by qRT-PCR and analyzed by 2^–ΔΔCt^ method using *β_ACTIN* (ACTB) as normalizing control. Data shown are the mean ± SD. *P* value was obtained by Student’s *t*-test. *: *P*<0.05, **: *P*<0.005.

**Figure S10: Quantification of isolated bone marrow cells with ATM mediated γH2AX (pS139) DNA damage as depicted in Figure 2c.**

Raw data for the quantification of isolated bone marrow cells with ATM mediated γH2AX (pS139) DNA damage signal at the indicated time after 2 Gy IR as depicted in Figure 2c. C, non-irradiated control.

**Figure S11: Enhanced stabilization of p53 and γH2AX in *Aplf^-/-^* cells after IR exposure.**

Isolated bone marrow cells were harvested and examined by western blot analysis at the depicted time intervals post IR at 2Gy ex vivo. Antibodies used were: anti-histone H2A.X (Millipore, catalogue No. 07-627), Anti-H2AX (pS139) (BD -Pharmingen, catalogue No.560443), Anti-p53 (CM5) (Leica Biosystems, catalogue No. NCL-p53-CM5p), and anti-β-actin (C4, Santa Cruz Biotechnology, catalogue No. sc-47778).
